# Salinomycin-Based Drug Delivery Systems: Overcoming the Hurdles in Cancer Therapy

**DOI:** 10.3390/pharmaceutics13081120

**Published:** 2021-07-22

**Authors:** Lucia Ruxandra Tefas, Cristina Barbălată, Cristian Tefas, Ioan Tomuță

**Affiliations:** 1 Department of Pharmaceutical Technology and Biopharmacy, Faculty of Pharmacy, “Iuliu Hațieganu” University of Medicine and Pharmacy, 41 Victor Babeș Street, 400012 Cluj-Napoca, Romania; tefas.lucia@umfcluj.ro (L.R.T.); barbalata.cristina@umfcluj.ro (C.B.); tomutaioan@umfcluj.ro (I.T.); 2 Department of Gastroenterology, “Prof. Dr. Octavian Fodor” Regional Institute for Gastroenterology and Hepatology, 19–21 Croitorilor Street, 400162 Cluj-Napoca, Romania; 3 Department of Internal Medicine, “Iuliu Hațieganu” University of Medicine and Pharmacy, 8 Victor Babeș Street, 400012 Cluj-Napoca, Romania

**Keywords:** salinomycin, nanoparticles, cancer stem cells, cancer, drug delivery system, nanotechnology, nanosystems

## Abstract

Cancer stem cells (CSCs) are reportedly responsible for the initiation and propagation of cancer. Since CSCs are highly resistant to conventional chemo- and radiotherapy, they are considered the main cause of cancer relapse and metastasis. Salinomycin (Sali), an anticoccidial polyether antibiotic, has emerged as a promising new candidate for cancer therapy, with selective cytotoxicity against CSCs in various malignancies. Nanotechnology provides an efficient means of delivering Sali to tumors in view of reducing collateral damage to healthy tissues and enhancing the therapeutic outcome. This review offers an insight into the most recent advances in cancer therapy using Sali-based nanocarriers.

## 1. Introduction

Cancer is one of the leading causes of death worldwide, and is considered a complex condition since a large number of factors contribute to its onset and progression [1]. Recent studies have revealed that tumors are heterogeneous in nature and contain a small subpopulation of cells capable of self-renewal, unlimited proliferation, and differentiation into other cell types, named tumor-initiating cells (TICs) or cancer stem cells (CSCs) [2]. There is evidence that CSCs are in part responsible for resistance to chemotherapy and radiation therapy, and show an increased metastatic potential. These properties play a pivotal role in promoting tumor growth, progression, and recurrence [2,3].

It has become evident that the suppression of highly proliferating differentiated tumor cells by conventional therapeutic approaches, without the concomitant restriction of the more quiescent CSCs, does not lead to complete eradication of the tumor [4]. On the contrary, surviving CSCs can regenerate the tumor mass into a more aggressive malignancy, causing relapse and subsequent treatment failure [5]. Thus, CSCs have been a major focus in oncology in recent years and may be a target for successful cancer therapy. That is why the latest research has focused on identifying new effective ways to eliminate CSCs. A breakthrough was made in 2009 with salinomycin (Sali), a monocarboxylic polyether antibiotic isolated from *Streptomyces albus*, intensively used in veterinary medicine for its anticoccidial and growth-promoting activity. Of 16,000 screened compounds, Sali was among a few to exhibit anti-CSC effects [2,6]. Its ability to selectively kill CSCs was first demonstrated in breast cancer, and further corroborated in other types of malignancies including prostate cancer, gastric cancer, pancreatic cancer, colorectal cancer, lung adenocarcinoma, osteosarcoma, squamous cell carcinoma, and leukemia [7,8]. Moreover, Sali has shown effectiveness against chemo-resistant cancer cells, and sensitizing effects in radio-resistant cancers [9].

Despite the compelling evidence that Sali acts against cancer cells, more importantly CSCs, it possesses unfavorable properties which greatly restricts its use in humans. Firstly, Sali and its sodium salt are lipophilic, and thus practically insoluble in water [10]. Secondly, even though several reports have verified the lack of toxicity in normal cells, Sali could induce muscular and neural toxicity in high concentrations [5,9]. To overcome these drawbacks, many researchers have resorted to formulate Sali in delivery systems capable of increasing the accumulation in tumor tissues through the enhanced permeability and retention (EPR) effect or active targeting.

The selection and design of the drug delivery system are of utmost importance since their properties, such as size and surface type, greatly impact the encapsulation of the drug, its pharmacokinetics and release profile, its biological fate after administration, and lastly the therapeutic effectiveness. The type of nanoparticle is mainly selected based on the physicochemical features of the drug of interest, most poorly soluble drugs usually being incorporated into liposomes, micelles, and polymeric nanostructures [11]. However, issues related to the potential toxicity of the carriers cannot be dismissed, and for this reason the majority of drug delivery nanosystems are constructed from biodegradable and biocompatible materials. It is noteworthy that the efficiency of most nanocarriers is correlated with particle size. It is generally accepted that small particles show increased bioavailability and accumulation in target cells. Furthermore, tailoring of surface properties through modifications with hydrophilic polymers, targeting ligands or other agents is another way of optimizing the nanosystem in terms of stability, circulation time, and efficacy [12]. Consequently, the judicious selection of materials and manufacturing method together with a rigorous definition of the objectives and quality characteristics allow to generate a successful drug delivery system.

This review offers an overview on the developed drug delivery systems based on Sali, and brings forth the recent advances in cancer and CSC research. Although not exhaustive, to our knowledge, this is the first detailed exposition of the various Sali nanoformulations, underscoring their potential in targeting CSCs, and also highlighting the role and importance of the nanoformulation in achieving therapeutic efficacy.

## 2. Cancer Stem Cells

Cancers are made up of multiple clonal populations of cells that differ among themselves in many aspects, including karyotype, growth rate, immunological characteristics, and expression of surface markers [13]. CSCs are a subpopulation of cells within a tumor and have typical characteristics related to somatic stem cells, such as the ability to self-renew and differentiate into diverse specialized cell types [14].

Since they were first identified, their origin has been the source of an ongoing debate. Exposure to toxins, mutations, metabolic shifting, cellular plasticity, cell fusion, horizontal gene transfer, and de-differentiation have all been suggested as possible etiological factors of CSCs [15]. Given that some CSCs have phenotypes, functions, and surface receptors resembling normal stem cells, it has been postulated that CSCs might develop from these secondary to genetic mutation or environmentally induced changes. Another theory is that CSCs arise from normal somatic cells which gain stem-like features through processes such as epithelial–mesenchymal transition (EMT). EMT is a process that allows a polarized epithelial cell to undergo several biochemical changes that enables it to assume a mesenchymal cell phenotype, which includes an enhanced migratory capacity, invasiveness, and an increased resistance to apoptosis [16]. This is also one of the fundamental processes that give CSCs their ability to form metastases.

CSCs are involved in the development and growth of tumor masses. When transplanted in mice with compromised immune systems, CSCs can reform a tumor identical to the one they have been isolated from. Moreover, they are involved in metastatic dissemination. CSCs are tumorigenic because they can generate all cell types found in a particular tumor, being thus responsible for their heterogeneity. They can even trans-differentiate to the vascular endothelium forming blood vessels [17,18].

CSCs can be identified and isolated from solid and hematological tumors using techniques such as fluorescence-activated cell sorting (FACS), which is a specialized type of flow cytometry. Some of the markers frequently used for CSC isolation include CD133, EpCAM, CD90, CD44, CD24, CD34, CD200, and ALDH1A1 [19].

Two key properties of CSCs that give them resistance to conventional chemotherapy and irradiation are self-renewal and quiescence. CSCs’ ability to self-renew and differentiate is kept under control with the help of multiple regulatory networks and signaling pathways, including the Hedgehog, Notch, Wnt/β-catenin, PTEN, and BMI1, as well as micro-RNA (miRNA) circuits [14,20]. Cellular quiescence means that cells are recruited into the G0-phase of the cell cycle but remain capable of cell division in response to mitotic stimulation [21]. Molecules involved in the regulation of CSC quiescence include tumor suppressors p53 and RB, cyclin-dependent protein kinase inhibitors p21, p27, and p57, and a number of miRNAs [22]. Usually, chemotherapeutic agents target highly proliferative cells through DNA damage and inhibition of mitotic division. However, their action is limited in regard to slow and non-dividing cells such as CSCs [23]. This could also explain the fact that after initial reduction of the size of the tumor mass, surviving CSCs can repopulate the tumor, giving rise to relapses.

In addition, CSCs express high levels of transmembrane transport proteins such as ATP-binding cassette transporters, which allow an increased efflux of chemotherapeutic agents out of the cells [23]. These proteins are usually located on the cell membrane and have the ability to export doxorubicin and methotrexate, among others [24]. CSC-mediated therapy resistance is also associated with avoidance of apoptosis through the rho family of proteins, non-coding RNAs, as well as stem cell niches—areas of tissue that provide specific microenvironments, which maintain and promote the CSCs’ capacity to self-renew and to generate differentiated progenies [23,24].

Furthermore, CSCs can evade the immune system using surface transmembrane glycoproteins which inhibit macrophage phagocytosis. One of the most studied such molecule is CD47 which is overexpressed in many cancers. The interaction between CD47 expressed on the surface of CSCs and signal regulatory protein α (SIRPα), which is abundant in myeloid cells, acts as a “self” signal leading to suppression of macrophages and dendritic cells [25,26].

Given these abilities of CSCs to escape destruction by the immune system or with conventional oncological therapy, alternative therapies that act on other mechanisms are needed.

## 3. Salinomycin (Sali)

Sali is a polyether monocarboxylic acid produced by *Streptomyces albus*, classically used as an agricultural antibiotic to prevent coccidiosis in chickens [27]. It exhibits high antimicrobial activity against Gram-positive bacteria, *Clostridium perfringens*, mycobacteria, and some filamentous fungi [28].

**Figure 1:**
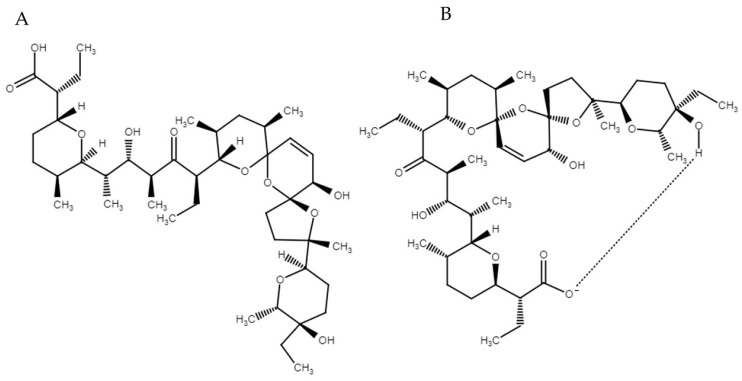
Chemical structure of salinomycin [5,31] in native form (**A**) and in pseudocyclic form (**B**) (Figure created with chem-space.com [accessed on 20 June 2021]).

Sali’s physicochemical properties are related to its structure (Figure 1), and several functional groups have been identified as essential for its biological effects, i.e., tetrahydropyran and tetrahydrofuran rings, carboxylic acid, hydroxyl groups, and ketones. Additionally, it contains a complex tricyclic bis-spiroketal system which imparts rigidity to the molecule. In total, Sali’s molecule contains 18 chiral centers. In native form, the molecule has an open structure (Figure 1A), but has the ability to form “head-to-tail” intramolecular hydrogen bonds between the carboxylic moiety on one end of the molecule and the hydroxyl group at the other end of the molecule. As a result, the molecule adopts a pseudocyclic structure (Figure 1B) in which the oxygen atoms face towards the interior of the structure, and thus being able to bind metal ions. Because the outer backbone is hydrophobic, the molecule is not soluble in polar media, and can easily cross lipophilic membranes [5,29,30].

**Figure 2:**
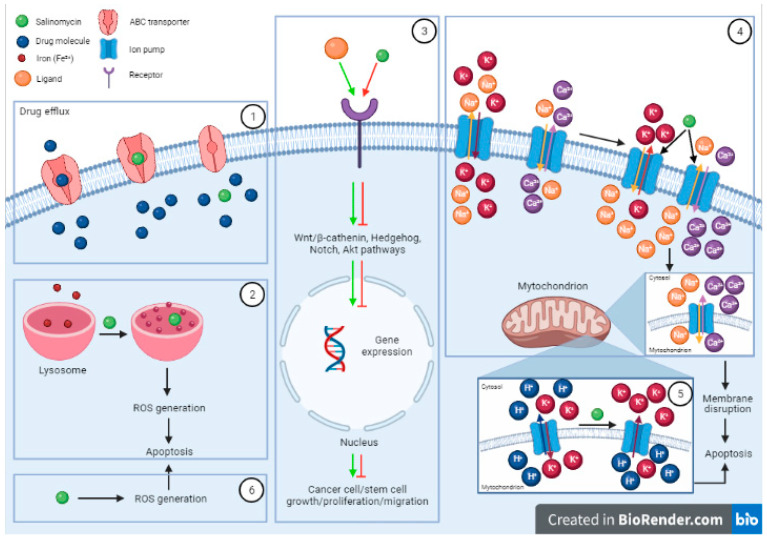
The mechanism of action of salinomycin: (**1**) Interfering with ABC transporters; (**2**) Accumulation and sequestration of iron in lysosomes; (**3**) Interfering with the Wnt/β-catenin, Hedgehog, Notch, and Akt signaling pathways; (**4**) and (**5**) Interfering with ion pumps; (**6**) Induction of reactive oxygen species (ROS) (Figure created with BioRender.com [accessed on 25 June 2021]).

In a study published in 2009 that evaluated 16,000 chemical compounds for their selective toxicity on breast CSCs, Gupta et al. showed that Sali reduced the proportion of CSCs by more than 100-fold relative to paclitaxel [32]. The way it seems to kill CSCs is through multiple mechanisms (Figure 2) which include interfering with ABC-binding transporters, the Wnt/β-catenin, Hedgehog, Notch, and Akt signaling pathways as well as mitochondrial function [8,33]. Sali has an affinity predominantly to sodium and potassium ions. In CSCs it induces the efflux of intracellular K^+^, an increase in intracellular Ca^2+^ and subsequent reduction of intracellular pH [34]. In addition, it leads to accumulation and sequestration of iron in lysosomes. Secondary to this, cells trigger the degradation of ferritin in lysosomes, leading to formation of reactive oxygen species. This in turn promotes lysosomal membrane permeabilization and activates apoptosis [35]. Sali has been shown in different in vivo and in vitro studies to be effective in eradicating CSCs, therefore reducing tumorigenicity [27,36,37,38,39,40].

However, some of the limits of Sali are its hydrophobicity and toxicity during systemic administration. Sali and its sodium salt have a solubility in water of less than 0.1 mg/mL, thus being considered practically insoluble. Since solubility is a crucial physicochemical property of drugs, a poor solubility in aqueous media results in low absorption from the active site and consequently poor bioavailability [6,41]. In veterinary medicine and animal husbandry, Sali-containing granules (Bio-cox, Sacox) are mixed with animal feed before use [5]. In previous studies, Sali has been dissolved in ethanol or 1% DMSO mixture and administered by injection to animals [32,42]. However, the toxicity of such solvents raises safety issues. Another important restriction in the use of Sali in clinical practice is its serious toxicity. It has been demonstrated that Sali exhibits severe toxicity on nervous cells (dorsal root ganglia and Schwann cells) at concentrations at which it is cytotoxic against CSCs [6]. It is believed that Sali induces peripheral neuropathy by interfering with the Na^+^/Ca^2+^ exchangers in the plasma membrane and mitochondria. In addition, Sali can cause acute cardiac and skeletal muscle degeneration and necrosis possibly through the same mechanisms which involves an elevation in cytosolic Ca^2+^ and Na^+^. Furthermore, this effect is also mediated by lipid peroxidation of cell membranes leading to membrane damage and subsequently necrosis [43]. However, Sali’s toxicity in mammals remains to be explained. Interestingly, it appears that the toxic effects depend on the species, which explains why chicken and cattle are the least sensitive to Sali, while horses and dogs are increasingly susceptible [6]. The clinical signs of ionophore (including Sali) intoxication reported for different animal species include anorexia, diarrhea, hypoactivity, depression, leg weakness, ataxia, and recumbency. Sali has been reported to induce neural toxicity in cats. High mortality was reported in turkeys after ingestion of Sali in feed. Acute toxicity studies have been carried out for Sali on different animal species. The median lethal dose (DL_50_) for Sali varies from 40 mg/kg in chicken to 57.4 mg/kg in mice. Unfortunately, since no antidote is available to counteract Sali’s toxic effects, treatment is mostly supportive. However, in one study in pigs, toxicity symptoms induced by Sali partly subsided after the administration of vitamin E [44]. However, Sali’s toxicity in humans is scarcely documented in the literature. Scherzad et al. showed that 10–20 µM Sali induced cytotoxicity in human nasal mucosa cells and peripheral lymphocytes in vitro. In another study, the same research group revealed that 24 h exposure of human mesenchymal stem cells to Sali affected the cell viability in a dose-dependent manner, but without affecting their ability to differentiate into adipocytes and osteocytes. Chronic exposure to Sali did not alter the mesenchymal stem cells’ pluripotency, but a decrease in their migration ability was observed [45]. In a case report from a clinical pilot study on a patient with metastatic invasive ductal breast cancer, the administration of 200 µg/kg Sali intravenously every other day produced a significant regression of the metastases. In another study, a patient with advanced and metastatic squamous cell carcinoma of the vulva, received the same dose of Sali in combination with erlotinib. Due to the severe adverse effects of erlotinib, the patient continued the monotherapy with 250 µg/kg Sali given intravenously every second day which stabilized the malignancy. In both cases, Sali induced minor side effects such as tachycardia and mild tremor, with no long-term toxicity [8]. In contrast, one case report presented the threatening side effects of Sali. The accidental ingestion and inhalation of approximately 1 mg/kg Sali by a farmer making animal feed mixes, led to severe side effects, including leg weakness, nausea, photophobia, prolonged pain, and rhabdomyolysis, resulting in a six-week hospitalization [46]. According to a risk assessment report by the European Food and Safety Authority, the acceptable daily intake of salinomycin for humans was set at 5 µg/kg [29].

Therefore, in order to increase the solubility and tumor delivery rates and reduce the side effects, nanocarriers could be used for targeting and delivery of Sali to tumor sites. The incorporation of Sali in nanoparticles significantly improves its pharmacokinetic profile. Sali has been shown to be poorly absorbed after oral administration, resulting in low bioavailability and high variability in plasma concentrations. It is quickly metabolized by CYP enzymes, especially CYP3A4, and has a high intrinsic clearance. Moreover, it extensively binds to plasma proteins, which could account for the low clearance observed in vivo [47]. Several reports on the pharmacokinetics of Sali-loaded nanoparticles in animals show a prolongation of the elimination half-life with a concomitant reduction in clearance. In addition, compared to the free drug, the area under the curve (AUC) is significantly increased for nanoparticle formulations of Sali. These findings suggest that nanoparticles prolong the circulation of Sali and increase its concentration in the blood [48,49,50]. Moreover, another pharmacokinetic parameter, namely the volume of distribution (Vd) seems to be affected. Since Vd indicates the theoretical volume in which the drug is distributed after administration, lower Vd values observed for Sali-loaded nanoparticles suggest that Sali is mainly retained in the plasma [42,48,50]. Therefore, this strategy allows to employ the EPR effect for the drug to accumulate in tumor tissue. At the same time, it reduces the accumulation of Sali in other tissues, i.e., lung or heart, which is susceptible to Sali’s toxicity, as mentioned before and evidenced in studies. However, Sali is distributed in other organs such as liver, spleen, kidneys to various extents [42,48,49]. It has been reported that a dose of 2 mg/kg Sali induces significant changes and inflammatory cell infiltration in lung, heart muscle, liver, and spleen in mice [51]. Moreover, doses of 8–10 mg/kg Sali in free form have been proven lethal to mice [51,52]. Thus, incorporation of Sali in nanoparticles can reduce its side effects. Since most types of nanoparticles are manufactured from GRAS materials approved by regulatory agencies for use in humans, they do not induce any significant toxic effects. Furthermore, owing to the preferential accumulation of the Sali-loaded nanoparticles at tumor sites, collateral toxicity to healthy tissues is minimized, and the treatment is well-tolerated by animals as reflected by the lack of behavioral changes (activity, appetite) or of the body weight [50,51,53,54].

**Figure 3:**
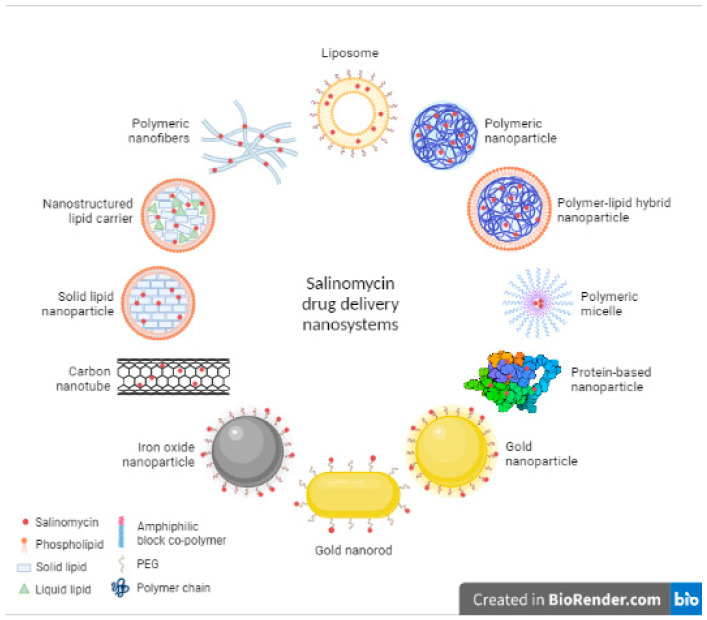
Drug delivery systems for salinomycin (Figure created with BioRender.com [accessed on 25 June 2021]).

**Figure 4:**
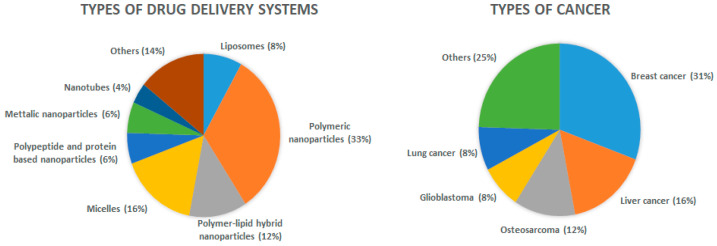
A summarized representation of the types of nanosystems (**left**) used in various cancer models (**right**), indicated as percentages of the studies reviewed herein.

Several nanoparticle formulations such as liposomes, polymeric nanoparticles, micelles, and metallic nanoparticles have been reported as drug delivery carriers for Sali (Figure 3) in various types of cancer, and are reviewed in this article (Figure 4). This strategy seems to prolong drug circulation time, increase tumor targeting, and potentiate Sali’s therapeutic effect.

However, since each type of nanoparticle possesses different properties regarding drug delivery, toxicity, and biopharmaceutical properties, the selection of a suitable type of nanoparticle to deliver Sali should be made taking into account the advantages and disadvantages of each type of nanoparticle (Table 1).

## 4. Types of Drug Delivery Systems for Salinomycin (Sali)

### 4.1. Liposomes

Liposomes are spherical vesicles composed of one or more phospholipid bilayers surrounding aqueous spaces. They have been extensively investigated and used in nanomedicine, especially in oncology, due to their high biocompatibility, ease of manufacturing, favorable pharmacokinetic profile, and easy surface tailoring. To prolong the systemic circulation time, liposomes are usually modified with polyethylene glycol (PEG) [66]. The hydrophobic nature of Sali makes it a suitable candidate for incorporation into the phospholipid bilayer of liposomes (Table 2).

It has become agreed upon that for a successful anticancer therapy both the CSCs and the bulk tumor cells must be eliminated, since CSCs have the ability to restore the tumor mass [66]. In order to achieve this, a common approach is to combine two anticancer agents that selectively target CSCs and non-CSCs, respectively. Therefore, Sali is generally associated with a conventional chemotherapeutic drug. However, certain types of malignancies are resistant to chemotherapy, which in most cases leads to recurrence and metastasis. Doxorubicin is largely used as a chemotherapy drug in various types of cancer, especially breast cancer, and moreover it can induce drug resistance leading to poor prognosis. Due to differences in hydrophilicity, both drugs can be successfully incorporated into liposomes: Sali, as previously mentioned, in the phospholipid membrane, while doxorubicin in the aqueous core of the liposome. Maintaining a synergistic drug ratio between the co-loaded drugs is crucial for achieving therapeutic efficacy, therefore several factors must be taken into account in co-delivery: optimum entrapment of each drug, controlled release after administration, and similar if not identical delivery times [67].

Kim et al. developed a nanoplatform for eradicating breast CSCs and non-CSCs by co-encapsulating Sali and doxorubicin in cross-linked multilamellar liposomes. The liposomes were prepared by the conventional dehydration-rehydration technique using 1,2-dioleoyl-sn-glycero-3-phosphocholine (DOPC), 1,2-dioleoyl-sn-glycero-3-phospho-(10-rac-glycerol) (DOPG) and 1,2-dioleoyl-sn-glycero-3-phosphoethanolamine-N-[4-(p-maileimidophenyl)butyramide] (MPB-PE). The resulting vesicles were fused in the presence of MgCl_2_ and further crosslinked with dithiothreitol (DTT). In order to prolong the circulation time, the liposomes were PEGylated. The in vitro assays on murine (4T1, 4T1D) and human (MDA-MB-231) breast cancer cell lines and in vivo study in 4T1 tumor cells-bearing mice indicated the superior cytotoxic effect of the co-loaded liposomal formulation on breast CSCs and cancer cells compared to the single-loaded liposomes and their physical association. The effective targeting of breast CSCs was validated by using putative breast CSC markers. This study demonstrated that the co-delivery of Sali and doxorubicin in a 5:1 synergistic drug ratio could enhance the cytotoxic potential against breast cancer by controlling the pharmacokinetics and distribution in vivo [67]. The same drug association was incorporated in liposomal vesicles, and evaluated against liver cancer, in a study conducted by Gong et al. The liposomes were manufactured from hydrogenated soybean phospholipids (HSPC), cholesterol, and 1,2-distearoyl-sn-glycero-3-phosphoethanolamine-*N*-[methoxy(polyethylene glycol)-2000] (DSPE-PEG2000) in a ratio of 85:10:5 using the lipid film method. The size of single drug-loaded liposomes and the co-loaded liposomes was around 100 nm, with a relatively narrow size distribution (polydispersity index (PDI) approximately 0.2), and a Zeta potential ranging from −30 mV to −40 mV, indicating a good stability. The liposomes encapsulating Sali and doxorubicin exhibited a prolonged half-life and decreased clearance in vivo, suggesting that PEGylation improves the passive targeting through the EPR effect. The combination of single-loaded liposomes and the co-loaded liposomes showed higher tumor inhibitory effects, and decreased the percentage of liver CSCs to a higher extent compared to the association of free Sali and doxorubicin, in liver tumor-bearing mice. Notably, the co-loaded liposomal formulation could maintain the synergistic drug ratio between 1:1 and 1:3 necessary for an efficient therapeutic outcome [66].

Since Sali acts preferentially on CSCs, and non-CSCs have the ability to spontaneously convert to CSCs, a potential strategy is to combine Sali with a sensitizing agent which could increase the sensitivity of cancer cells to therapy. Antimalarial agent chloroquine has been shown to repress autophagy by inhibiting lysosomal activity. The above-mentioned liposomal formulation was processed by the ethanol injection method according to the research of Xie et al. Chloroquine was actively loaded into the liposomes using the ammonium sulfate gradient method, and used as a sensitizing agent to increase the therapeutic efficacy of Sali towards liver cancer cells. The molar ratio between Sali and chloroquine was optimized at 1:5 to achieve synergistic effects. All resulting liposomes were around 120 nm in size, relatively monodisperse, with acceptable encapsulation efficiency and drug loading (around 70% and 3%, respectively). The co-loaded liposomes and combination of single-loaded liposomes induced significant cytotoxicity, apoptosis, and decrease in colony formation in HepG2 cells, compared to monotherapy with liposomal Sali. Chloroquine being an autophagy inhibitor could significantly increase the cytotoxicity of Sali in HepG2 cells when combined. However, these effects were not significant in HepG2 CSC-rich cells [68].

The remarkable anticancer properties of Sali have prompted scientists to develop more active or safer and better-tolerated derivatives. Given the affinity of Sali for monovalent ions, especially potassium, recent research has focused on synthesizing novel metal coordination compounds in view of increasing the therapeutic activity of Sali. Thereby, Momekova et al. successfully synthesized four different Sali complexes with potassium, nickel, manganese, and cobalt ions, and alongside the sodium salt loaded these metal compounds in sterically stabilized liposomes and evaluated their cytotoxic potential against a panel of three hematological cancer cell lines (KG-1, U-266 and Reh). The liposomes were prepared from DPPC, cholesterol and DSPE-PEG2000 by the conventional film hydration method. Due to differences in molar mass between the monovalent and divalent metal species, the optimal drug to DPPC ratio was found to be 0.5:1 and 0.1:1, respectively, which allowed the formation of unilamellar vesicles with sizes ranging between 130 nm and 160 nm, and uniform size distribution (PDI between 0.06 and 0.1). The inclusion of metal coordination compounds imparted a positive surface charge which proved to be in part responsible for the biological effects observed. In terms of cytotoxicity, the divalent metal compounds (in particular the manganese compound, followed by the cobalt and nickel complexes) proved to be more potent than the potassium and sodium salinomycinates, and furthermore, the incorporation into liposomes led to similar or even superior effects compared to the respective free form. It was demonstrated that the antitumor effects of the metal species in free or liposomal form is attributed to the induction of apoptosis and cell cycle arrest in the malignant cells [4].

Since their discovery more than 50 years ago, liposomes have been extensively investigated, and some formulations even reached approval for cancer therapy. Their appeal as drug delivery systems also stems from their resemblance to the structure and composition of cellular membranes. However, issues related to the long-term stability of liposomes have proven to be challenges relatively difficult to tackle. Although the number of studies involving the use of liposomes as drug delivery systems for Sali is quite limited, the previously-described research validates PEGylated liposomes as effective vehicles for the delivery of Sali to cancer cells. It seems that liposomal combination therapy is preferred to single drug delivery since this strategy increases the drugs’ anticancer effects by synergism, while reducing the side effects. Furthermore, liposomal co-delivery of Sali with a conventional anticancer drug appears to be a more effective approach since it allows the eradication of both CSCs by Sali, and the bulk tumor cells by the chemotherapeutic agent. Moreover, other advantages of using liposomes for co-delivery are the feasibility of encapsulating both lipophilic and hydrophilic drugs in the same carrier, and the convenient single administration protocol. Therefore, the liposomal co-delivery allows the synchronized delivery of the payloads to the target site.

### 4.2. Polymeric Nanoparticles

Polymeric nanoparticles are particles with sizes in the nanometer range which encompass both nanocapsules and nanospheres, distinguished based on their morphology. While nanocapsules are reservoir-type systems, nanospheres are matrix-type systems. Nanocapsules generally contain an oily core enveloped in a polymeric shell which controls the release rate of the drug. In this case, the drug is usually dissolved in the core. Nanospheres, on the other hand, are composed of a continuous, uniform polymeric network, in which the drug can be entrapped, or it can be adsorbed on the surface of the particles [69].

The safety of nanoparticulate drug delivery systems is an important issue, and therefore the general approach is to use biodegradable and biocompatible polymers. Various approved such polymers are available, but the most commonly employed polymeric materials in nanoparticle development are poly(lactic acid) (PLA), poly(lactic-co-glycolic acid) (PLGA), and poly(Ɛ-caprolactone) (PCL) [54]. The polymeric nanoparticles developed for the delivery of Sali are summarized in Table 3.

Wang et al. developed gelatinase-responsive polymeric nanoparticles by inserting the Pro-Val-Gly-Leu-Iso-Gly (PVGLIG) gelatinase-cleavable peptide between PCL and PEG chains in order to selectively deliver Sali to gelatinase-rich tumor sites. The research group investigated the optimum method for preparing the Sali-loaded core–shell nanoparticles by employing two preparation techniques, namely the nanoprecipitation and single emulsion methods. According to their results, the nanoparticles showed a mean size of around 150 nm and 230 nm, respectively. It was reported that a 1% concentration of Pluronic F68 as stabilizer yielded particles with lowest size and highest stability. However, the single emulsion method led to higher Sali entrapment efficiency (89.7% vs. 81.51%), superior stability, and more sustainable drug release (70% vs. 80% after 24 h), which suggests that this method could be a suitable one for encapsulating Sali into stimuli-responsive polymeric nanosystems. Furthermore, the in vivo toxicity study indicated a higher survival rate of mice treated with Sali-loaded nanoparticles vs. non-entrapped drug. This suggests that incorporating Sali into nanoparticles can reduce its side effects by increasing its concentration in tumors and limiting the exposure of normal tissues [70]. In a subsequent study, the same research group demonstrated the anticancer effects of the intelligent Sali-loaded gelatinase-responsive nanosystem against HeLa cervical cancer cells. The antitumor effect was explained by Sali’s ability to induce apoptosis and inhibit the proliferation of cervical CSCs in vivo, as evidenced by the up-regulation of caspase-3 and down-regulation of PCNA and Ki-67. Furthermore, the Sali-loaded nanoparticles decreased the expression of CD44 and CD133, and reduced the tumor seeding ability and tumor growth rate in tumor-bearing mice, compared to the free drug, suggesting the cervical CSC targeting ability of Sali. In addition, Sali-loaded nanoparticles reduced the expression of VIM and increased the expression of E-cadherin, suggesting that Sali exhibits inhibitory effects on cervical CSCs by targeting the ZEB1 and ZEB2 pathway, thus inhibiting the EMT process [51].

In a recent study, Mineo et al. developed a novel gel permeation chromatography (GPC) method for determining the encapsulation efficiency and drug loading of Sali in PLA nanoparticles. The polymeric nanoparticles were obtained by nanoprecipitation, and were further functionalized with folic acid by click chemistry. The developed GPC technique revealed efficient encapsulation (98–99%) and drug loading (8.8–8.9%) of Sali, subsequently corroborated by voltametric analyses. Interestingly, the binding of folic acid to the surface of the nanoparticles drastically increased their size from around 100 nm to over 600 nm. The inclusion of Sali in PLA nanoparticles did not alter the drug’s biological effect, exhibiting cytotoxicity against MG-63 osteosarcoma cells and osteospheres similarly to the free drug. However, functionalization with folic acid showed no obvious benefit compared to non-decorated nanoparticles [71].

Irmak et al. demonstrated the superior efficacy of Sali in osteosarcoma, after encapsulation in PLGA nanoparticles by emulsion-diffusion-evaporation. The polymeric nanoparticles were slightly large (around 188 nm), but monodisperse, and extremely stable. Compared to the more frequently employed PLGA copolymer with 50:50 ratio between the polylactic and polyglycolic segments, Irmak et al. used PLGA 65:35 which enabled the encapsulation of a higher amount of Sali (97%). An initial burst of Sali from the nanoparticles ensured a rapid and effective cytotoxic effect towards MG-63 osteosarcoma cells, while the subsequent gradual release of drug sustained this effect. Noteworthy, compared to the free form, the encapsulated Sali decreased the proliferation and increased the apoptosis of osteosarcoma cells more effectively, by inducing caspase-3 expression and suppressing the β-catenin and c-myc pathways [10].

In a study conducted by Aydin et al., the effects of Sali via polysorbate 80-coated PLGA nanoparticles were evaluated. Polysorbate 80 (P80) was chosen as stabilizer and coating agent due to its inhibitory effect on efflux proteins which are responsible for drug resistance and hamper drug delivery to the brain. The polymeric nanoparticles, prepared by the emulsion–solvent evaporation method, exhibited sizes ranging from 187 nm to 293 nm, and a significant increase in the average diameter was observed for P80-coated nanoparticles. Sali was loaded into the nanoparticles in a proportion of approximately 60%, and released in a sustained manner (around 90% cumulative released after 480 h). Coating with P80 strongly facilitated the uptake of Sali-loaded nanoparticles in glioblastoma cells (around 14% after 60 min) which is essential for accumulation in the brain tissue. The cellular viability of T98G glioblastoma cells was significantly reduced when treated with P80-coated nanoparticles as opposed to uncoated carriers, and important morphological changes were noted by fractured actin cytoskeleton due to cell apoptosis [72].

To improve the retention of nanoparticles at the tumor site, the current trend in nanotechnology is to use targeted nanoparticles for active targeting of tumors. This is achieved by attaching affinity ligands onto the surface of the nanoparticles for selective delivery of the payload to the target tumor site by specific interaction with the corresponding receptors expressed on the surface of the cancer cells. Targeted nanocarriers have been shown to be taken up by cancer cells more efficiently than their non-targeted counterparts, thus minimizing the side effects to other tissues [73]. Accordingly, Mi et al. used Sali-loaded PLGA-PEG nanoparticles for specific eradication of CD133^+^ CSCs through conjugation with CD133 antibody, in ovarian cancer. The polymeric nanoparticles increased the antitumor effect of Sali in ovarian cancer, and moreover, the antibody-modified nanoparticles were more capable of eradicating the ovarian CSC population than the controls, upon binding to the CD133 surface marker [74]. Furthermore, Ni et al. showed that aptamers-targeted nanoparticles could specifically deliver Sali to CD133^+^ osteosarcoma CSCs in vitro and in vivo. The Sali-loaded PEGylated PLGA nanoparticles were conjugated with A15 aptamers for selective targeting of CD133 antigen. The aptamers-targeted nanoparticles proved to be more effective in eradicating osteosarcoma CSCs than the non-decorated nanoparticles and the unentrapped drug, respectively [54]. Compared to the research of Ni et al., Jiang et al. demonstrated that the dual conjugation of polymeric nanoparticles with CD133 aptamers A15 and EGFR aptamers CL4 significantly improved the cellular recognition and antitumor activity of Sali in hepatocellular carcinoma [53]. In all above-described studies, the polymeric nanoparticles were obtained by an emulsion–solvent evaporation method. Regarding the characteristics of the nanoparticles, the average size was around 150 nm, exhibited a narrow size distribution (PDI around 0.2) and a negative Zeta potential (below −20 mV), and Sali entrapment efficiency was over 50%. A fast release of Sali (approximately 40–50%) from the PEGylated PLGA nanoparticles was observed in the first 24 h in all above-described studies, following a sustained release with a total release of around 80–85% Sali over a period of up to 12 days or more [53,54,74].

Conjugation with Herceptin (HER, Trastuzumab) improved the penetration of Sali-loaded PLGA nanoparticles in MCF-7 breast cancer cells which overexpress the HER2 receptor, compared to the non-targeted nanoparticles. Furthermore, an enhanced cellular uptake was observed at a higher Sali concentration which could be related to the amount of HER immobilized on the surface of the nanoparticles. Regarding the manufacturing of the nanoparticles, an emulsion–solvent evaporation method was applied using PLGA as the matrix-forming agent and didodecyl dimethyl ammonium bromide (DMAB) as stabilizer. Attachment of HER to the nanoparticles increased their size from around 200 nm to 257 nm, and also the size distribution. Sali was successfully loaded into the nanoparticles with an efficiency ranging from approximately 60% to 90%. Due to the hydrophobic nature of Sali, the release from the nanoparticles was prolonged; however, HER-immobilized nanoparticles displayed a faster release probably due to the hydrophilicity of HER. Overall, the research of Aydin et al. showed the potential of HER-decorated PLGA nanoparticles for targeted delivery of Sali to breast cancer cells [73].

It has become increasingly obvious that for a successful treatment, both CSCs and bulk cancer cells must be eradicated, as previously mentioned in this paper. Therefore, the combination of multiple anticancer drugs that target CSCs and non-CSCs, delivered simultaneously or in different carriers is gaining more ground. A summary on the combinatorial delivery of Sali and various anticancer drugs in polymeric nanoparticles is presented in Table 4. Li et al. addressed this issue by developing PLGA-PEG nanoparticles separately entrapping Sali and docetaxel, with small size (130–150 nm), good polydispersity (0.11–0.14), reasonable stability (−20 mV Zeta potential), high encapsulation efficiency (80%), and sustained release (around 80% after 108 h). The researchers found that Sali in free and encapsulated form selectively eradicated gastric CSCs, while docetaxel in free and encapsulated form mainly killed the bulk gastric cancer cells. However, the combination of nanoparticles entrapping Sali and docetaxel, respectively suppressed tumor growth more efficiently than the single drug-loaded nanoparticles or combination of free drugs [75]. In contrast, Gao et al. opted for the incorporation of the two drugs in the same nanoparticulate system for co-delivery in breast cancer. Apart from PLGA which was used as the building block for the nanoparticles, the researchers added TPGS to control the size of the particles, the drug encapsulation and release, and also as a potential inhibitor of the P-glycoprotein efflux pump. The co-loaded nanoparticles were prepared by nanoprecipitation, and exhibited a mean particle size of 73.83 nm, were monodisperse, had a Zeta potential of −25.7 mV, and had a satisfactory entrapment efficiency of 53.28% for Sali and 84.96% for docetaxel, respectively. According to the pharmacokinetic analysis, the entrapment in more rigid polymeric nanoparticles prolonged the circulation time and maintained the synergistic 1:1 ratio of both drugs in vivo for 24 h. In addition, the co-delivery proved more effective in tumor targeting, and eradicating both bulky breast tumor cells and CSCs than the single treatments or the combination of two distinct single drug-loaded nanoparticles [76]. Similarly, Li et al. encapsulated Sali in TPGS-emulsified PLGA nanoparticles, but as a means to increase the solubility and bioavailability of the drug after oral administration in nasopharyngeal carcinoma. Compared to the common intravenous administration, oral chemotherapy has the power to improve patient compliance, being a more convenient route of administration. Incorporation into orally administered TPGS-PLGA nanoparticles significantly improved the pharmacokinetics and absorption of Sali, which consequently improved the therapeutic performance in vivo. The improved oral bioavailability could be attributed to the small size of the nanoparticles (62.86 nm) which favored tumor cell uptake, and the negative surface charge (−28.7 mV) responsible for a high stability in the circulation. The entrapment efficiency (56.35%) and drug loading (4.79%), however moderate, ensured a sufficient dose of Sali for effective restraining of nasopharyngeal carcinoma stem cells [77]. Zhang et al. reported that a combination of Sali-loaded nanoparticles and gefitinib-loaded nanoparticles was more efficient in suppressing tumor growth both in vitro and in vivo that the free drugs combined or single therapy with drug-loaded nanoparticles. Both nanoparticles were obtained by emulsion–solvent evaporation which proved to be a good approach to incorporate the two hydrophobic drugs in high amounts (80% entrapment efficiency), leading to nanoparticles of 130–150 nm, with sustained release over 120 h. Compared to gefitinib or gefitinib-loaded nanoparticles alone, the combined treatment, whether in free form or incorporated into nanoparticles was able to reduce the percentage of CSCs in lung tumors from mice the most. Furthermore, the tumor volume and weight from A431 xenograft-bearing mice were significantly lower, while no body weight loss was recorder for this treatment group. These results underline the necessity of combining a chemotherapeutic agent with an anti-CSC drug for a better and well-tolerated anticancer therapy [78].

Muntimadugu et al. developed PLGA-based nanoparticles for the simultaneous delivery of Sali and paclitaxel in breast cancer. The nanoparticles were obtained by the emulsion solvent diffusion method using a cationic stabilizer, and showed a mean size below 150 nm. Coating the polymeric nanoparticles with hyaluronic acid for CSC-specific CD44 receptor targeting led to the highest cytotoxic effect with minimum IC_50_ values and enhanced cellular uptake in MCF-7 breast cancer cells, including CD44^+^ cells. In addition, a longer circulation time was achieved which demonstrated the improved bioavailability of the combination therapy when loaded into nanoparticles [79].

A combination of Sali and curcumin was loaded into PEG-PLGA copolymer nanoparticles functionalized with hyaluronic acid for specific targeting of breast CSCs. The nanoparticles were prepared by the double emulsion method using polyvinyl alcohol (PVA) as a stabilizer. The mean size of the particles increased after conjugation with hyaluronic acid from around 120 nm to 153 nm, and the surface charge was negative due to the carboxylic groups of hyaluronic acid. Sali and curcumin were encapsulated with an efficiency of around 70% and 82%, respectively. The release of Sali and curcumin from the nanoparticles was sustained, with a rapid release observed in the first hours. By conjugating CD44 glycoprotein-targeting moiety on the surface of the nanoparticles, the co-loaded delivery system exhibited enhanced cellular uptake, cytotoxicity, cell migration, and attachment inhibitory effects compared to the non-functionalized counterpart and single treatments. In addition, a molar ratio of 1:1 between Sali and curcumin promoted synergism against breast cancer. The hyaluronic acid-coupled nanoparticles promoted the G1/S cell cycle arrest, leading to subsequent apoptosis of breast CSCs. This suggests that hyaluronic acid-conjugated nanoparticles are a promising means of selectively delivering Sali and curcumin to breast CSCs [80].

Overall, polymeric nanoparticles have been extensively investigated as drug delivery systems for Sali. Compared to liposomes, polymeric nanoparticles have the advantage of possessing higher stability and a more controllable release pattern. However, when compared to liposomes, regardless of the preparation method, polymeric nanoparticles appear to be larger which could potentially hamper the uptake by cancer cells. The encapsulation of Sali in biodegradable FDA-approved polymers proved efficient in eradicating CSCs. Furthermore, surface modification with PEG ensures a prolonged circulation time and passive targeting ability by the EPR effect. On the other hand, active targeting by using ligands conjugated at the surface of the nanoparticles favors cellular uptake by receptor-mediated internalization, resulting in increased penetration in cancer cells. Other advantages of using functionalized polymeric nanoparticles for the delivery of Sali include increase of selectivity to specific cancer cells, and modulation of drug release. However, the development of polymeric nanoparticles for active targeting appears more complex, and the selection of an adequate targeting ligand mostly depends on the type of cancer and receptors expressed on the surface of cancer cells.

### 4.3. Polymer–Lipid Hybrid Nanoparticles

The main drawbacks associated with liposomes is their instability, unsatisfactory drug loading, and uncontrollable drug release [81]. However, liposomes are highly biocompatible and have easily tunable surface properties by coupling hydrophilic polymers such as PEG or other targeting moieties [82]. In contrast, polymeric nanoparticles have superior stability, drug-loading capacity, and more controllable drug release, but even when manufactured from biodegradable polymers, their biocompatibility does not equal that of liposomes. Polymer–lipid hybrid nanoparticles have emerged as an alternative to polymeric nanoparticles and liposomes, since these nanosystems combine the advantages and overcome the disadvantages of the two common types of drug delivery systems [81]. A summary of the research that investigated the anticancer effects of Sali-loaded polymer–lipid hybrid nanoparticles is included in Table 5.

The targeting ability and anticancer efficacy of Sali-loaded polymer–lipid hybrid anti-HER2 nanoparticles was investigated against breast CSCs and cancer cells in a study conducted by Li et al. A nanoprecipitation method was employed to prepare the hybrid nanoparticles using PLGA, soybean lecithin and DSPE-PEG2000. The nanoparticles were further conjugated with anti-HER2 Fab’ antibody for selective targeting of HER receptor which is known to be overexpressed in some breast cancers. The characterization of the hybrid nanoparticles revealed a mean size of 123.2 nm for the untargeted nanoparticles which slightly increased to 135.6 nm for the antibody-conjugated nanoparticles. The nanoparticle population was homogenous in size as indicated by the PDI of 0.2, and Sali was incorporated with 55% efficiency. The release pattern was biphasic, with an initial burst of around 50% in the first 12 h, followed by sustained release up to 96 h, with a cumulative percentage of 80% released Sali. The in vitro targeting ability of the Sali-loaded hybrid nanoparticles was investigated in two breast cancer cell lines, namely MDA-MB-361 and BT-474, in which aldehyde dehydrogenase (ALDH) was used as a breast CSC marker. The nanoparticles promoted the delivery of Sali to cancer cells, and the conjugation with anti-HER2 antibody further improved the targeting ability in both breast cancer cells and CSCs. Furthermore, anti-HER2 Fab’-decorated nanoparticles exhibited superior cytotoxic effects towards ALDH^+^ cells, suggesting that Sali preferentially eradicates breast CSCs in vitro. The Sali-loaded anti-HER2 Fab’-targeted nanoparticles reduced the tumorsphere formation and proportion of ALDH^+^ breast CSCs to a higher extent than the non-conjugated nanoparticles and unentrapped Sali, both in vitro and in vivo [81].

Melanoma is an aggressive type of skin cancer, and it has been demonstrated that CD20^+^ melanoma CSCs are pivotal for the initiation and metastasis of this malignancy. Therefore, eliminating CD20^+^ melanoma CSCs could ensure remission of the disease [83]. This theory was investigated by Zhang et al. who used ACD, an anti-CD20 DNA aptamer to promote specific and effective delivery of Sali to CD20^+^ melanoma CSCs. The presence of CD20 aptamers on the surface of the nanoparticles promoted the entry of Sali-loaded nanoparticles in CD20^+^, but not CD20^−^ A375 and WM266-4 melanoma cells, and enhanced the antitumor effect against melanoma CSCs in vitro and in tumor-bearing mice, compared to free Sali and non-conjugated nanocarriers, demonstrating the selective toxicity of CD20 aptamer-linked nanoparticles loaded with Sali towards CD20^+^ melanoma CSCs [83].

Conjugation of hybrid nanoparticles with CD44 antibody resulted in superior therapeutic efficacy against prostate CSCs than the non-linked nanoparticles and free Sali. In contrast to most of the described methods of polymer–lipid hybrid nanoparticle manufacturing which employed the one-step nanoprecipitation process, Wei et al. applied a two-step approach. Firstly, the PLGA nanoparticle core was obtained in the first step by an emulsion–solvent evaporation method, followed in the second phase by coating of the polymeric nanoparticles with a lipid shell (containing DSPE-PEG, phosphatidylcholine, and cholesterol) by using the conventional lipid film method. This approach allowed the formation of small size nanoparticles of approximately 130 nm, with negative surface charge, 75% Sali encapsulation efficiency, and sustained drug release (80% cumulative release) over 120 h [84].

Epidermal growth factor receptor (EGFR) is overexpressed in various types of cancers, and has been found to be overexpressed in CSCs as well, contributing to several characteristics of these TICs, including self-renewal and tumorigenesis. This suggests that EGFR could be a suitable target for numerous types of malignancies [85]. To test this hypothesis, Yu et al. developed EGFR aptamer-conjugated polymer–lipid hybrid nanoparticles and demonstrated their efficacy in targeting osteosarcoma cells and CSCs. The nanoparticles were prepared from soybean lecithin, DSPE-PEG and PLGA, and exhibited small size of below 100 nm, a negative Zeta potential of −20 mV, satisfactory encapsulation of Sali (around 65%), and a sustained drug release of 80% within 120 h. EGFR-immobilized Sali-loaded nanoparticles proved more effective in inhibiting the proliferation of U2O2 and MG-63 osteosarcoma cells and reducing the tumorsphere formation rate than the nontargeted nanoparticles and free Sali. Furthermore, the cytotoxic effect was increased towards CD133^+^ cells compared to CD133^−^ cells, suggesting that the hybrid nanoparticles preferentially eliminate osteosarcoma CSCs [85].

Similar hybrid nanoparticles were developed by Chen et al., and were conjugated with two ligands, namely CD133 and EGFR aptamers (CL4 and A15 aptamers, respectively), for the eradication of osteosarcoma cells and CSCs. As opposed to single targeting, dual targeting could address several cellular subpopulations overexpressing antigens. A superior cytotoxic effect against osteosarcoma Saos-2 and MG-63 cells and tumorsphere inhibitory effect were observed for the dual-targeted nanoparticles loaded with Sali compared to single-targeted, nontargeted nanoparticles or Sali alone. In addition, the dual-targeted nanocarrier inhibited tumor growth in vivo more successfully than the other counterparts. Therefore, conjugation with EGFR aptamers not only increased the efficacy of Sali-loaded nanoparticles against osteosarcoma cancer cells, but also against CD133^+^ osteosarcoma CSCs [86]. Similar findings have been reported by Zhou et al. for a dual-targeted hybrid nanocarrier composed of PLGA, phosphatidylcholine, cholesterol, and DSPE-PEG, for the delivery of Sali to lung cancer. Double conjugation with CD133 and EGFR aptamers promoted the entry of the Sali-loaded nanoparticles in H460 and A549 lung cancer cells and CSCs, achieving superior antitumor efficacy both in vitro and in vivo in tumor-bearing mice, compared to controls [82].

In summary, liposomes and polymeric nanoparticles have been combined into a single hybrid delivery system harboring the advantages of both types of carriers, such as small size of around 100 nm and high Sali incorporation efficiency, typical for liposomes, and good stability and sustained release of Sali of around 80% over an average period of 4–5 days, characteristic of polymeric nanoparticles. The hybrid nanoparticles have a polymeric core in which the drug is entrapped, and a lipid shell providing biocompatibility. Since Sali is hydrophobic in nature, good encapsulation can be achieved in the polymeric matrix of the hybrid nanoparticles. Additionally, a PEG coating provides steric stabilization and prolonged circulation in the bloodstream [87]. The hybrid nanoparticles were prepared using approved materials such as PLGA, phosphatidylcholine, cholesterol, and PEGylated DSPE. In most studies described above, the one-step procedure was preferred to manufacture the nanoparticles due to greater ease as opposed to the two-step approach which entails the separate preparation of the Sali-loaded polymeric core and lipid shell, respectively, followed by merger of the two. Furthermore, all investigated polymeric-lipid nanoparticles were conjugated with targeting ligands for selective binding of specific receptors which emphasizes the utility of and need for specific CSC-targeting strategies. Owing to endocytosis mediated by specific receptors expressed on the surface of cancer cells, targeted hybrid nanoparticles demonstrated better performance in vitro and/or in vivo, showing greater accumulation at tumor sites and enhanced cytotoxicity towards cancer cells.

### 4.4. Micelles

The low tumor-penetrating ability of nanoparticles, mainly due to their size, is a major obstacle in the successful delivery of anticancer drugs to tumor sites. Most nanoparticles which are larger than 50 nm accumulate around tumors primarily through the leaky vasculature of the tumor. On the contrary, particles with sizes below 50 nm have been shown to enter tumors more efficiently than their larger counterparts [89]. According to several reports, the hypoxic center and necrotic regions of tumors are rich in CSCs, therefore developing a drug delivery system with small size and enhanced penetration ability could facilitate the accumulation of anticancer drugs into the tumor [48]. Micelles are nano-sized self-assemblies of block copolymers with amphiphilic properties. In aqueous media, the hydrophobic segment faces the interior of the micelle, while the hydrophilic part forms an outer shell which protects and disperses drugs with poor solubility in water [90]. It is noteworthy that micelles could be designed to possess small sizes (around 10 nm) for a better penetration into solid tumors [89]. Furthermore, nanomicelles offer several advantages as drug delivery vehicles such as solubilization of lipophilic drugs in their inner hydrophobic core, high stability, prolonged in vivo circulation time, sustained drug release, and lastly their ability to passively target tumors through the EPR effect [91]. Several micellar formulations with Sali and their biological activity are described in Table 6.

Lipid-based micelles composed of DSPE-PEG2000 are of particular interest and have been exploited as drug delivery systems for Sali in several studies. For example, Zhu et al. developed such nanomicelles and used methotrexate not only as an anticancer drug, but also as a targeting ligand for specific binding to head and neck squamous cell carcinoma cells overexpressing folic acid receptors. The research group employed the classic lipid film method, which enabled to obtain small size particles of 15–20 nm, with uniform size distribution (PDI < 0.2), high stability (Zeta potential around −20 mV), and good encapsulation efficiency and drug loading for Sali of approximately 85% and 9%, respectively. Sali-loaded methotrexate-modified micelles were efficiently bound and taken up by head and neck cancer cells, leading to an efficient eradication of both CSCs and non-CSCs in vitro and in vivo, compared to the non-functionalized micelles and single or combined free drugs. Strikingly, the nanomicelles were well tolerated in mice and did not induce any major systemic toxicity, suggesting that the incorporation of Sali into micelles could significantly reduce the side effects of the free drug [89].

To enhance the delivery of Sali to cancer cells, some researches have focused on functionalizing nanomicelles with ligands for specific interactions with markers which are overexpressed in cancerous tissues. In this regard, Mao et al. developed internalizing RGD (iRGD) peptide-modified DSPE-PEG2000 micelles for the delivery of Sali to liver tumor. The small size of the lipid-based micelles (in the range of 13–14 nm) favored the internalization into HepG2 liver tumor cells and CSCs. In addition, the iRGD-conjugated micelles showed a high encapsulation efficiency (>90%), and released more than 60% of the incorporated Sali over 48 h. However, the cumulative release of Sali was greater (80% vs. 60%, respectively) at pH 5.5 than pH 7.4, which suggests that the release of Sali from the micelles is pH-dependent. The incorporation of Sali into lipid micelles enhanced its cytotoxicity towards liver tumorspheres, as well as bulk liver cancer cells, due to the selective toxicity of Sali on the CSC population. In addition, the iRGD conjugation approach proved effective, as the iRGD-modified micelles showed superior targeting ability and increased antitumor efficacy compared to non-conjugated micelles, both in vitro and in vivo. Furthermore, the conjugation prolonged the circulation time and increased the plasma concentration of Sali in rats, and showed no sign of systemic toxicity [48].

Some important issues concerning the use of peptides as targeting ligands include their immunogenicity, stability, and difficulty in binding to the nanoparticles [92]. Aptamers, which are short single-stranded oligonucleic acids, on the other hand, offer some advantages over peptide-based ligands, such as lack of immunogenicity and toxicity, lower molecular weight and possibility of synthesis with particular functional moieties for site-specific conjugation [92,93]. Accordingly, Leng et al. proposed that EGFR aptamers-modified Sali-loaded DSPE-PEG2000 nanomicelles could specifically target both lung CSCs and cancer cells overexpressing EGFR. Binding of CL4 aptamer to the micelles yielded particles of 24 nm, narrow size distribution and relatively low Zeta potential (around −20 mV). The cytotoxic effect of Sali towards CD133^+^ and CD133^−^ lung cancer cells was significantly enhanced by incorporation into micelles. Furthermore, EGFR aptamers-functionalized micelles proved more effective compared to non-targeted micelles both in vitro and in tumor xenograft-bearing mice. This suggests that the encapsulation in micelles, and particularly EGFR aptamers modification of micelles could efficiently mediate the uptake of Sali in EGFR-overexpressing lung cancer cells [93].

A major obstacle in developing drug delivery systems is the need for high amounts of carriers which can induce toxicity or side effects. However, if the drug delivery system is therapeutically active, the safety concern is greatly reduced. Taking advantage of this idea, Wang et al. used PEG-ceramide as the building block to fabricate a therapeutic drug carrier for Sali to liver cancer. Since ceramides have been reported to modulate cell death and cell cycle arrest in cancer cells, this approach allowed to achieve a synergistic effect of the drug and the carrier, at a molar ratio of 1:4. Due to its amphiphilic properties and low critical micellar concentration (CMC), PEG-ceramide formed micelles with spherical shape, small size (around 14 nm) and uniform dispersion. Sali was loaded into the micelles with 76.7% efficiency, and more than 50% was released over the course of 2 days, but differentially, as a function of pH. Compared to free Sali and DSPE-PEG micelles, PEG-ceramide micelles promoted the accumulation of Sali in liver cancer cells to a greater extent, and demonstrated superior cytotoxic effects in vitro. Interestingly, the PEG-ceramide nanocarrier significantly increased the apoptotic events in HepG2 liver cancer cells, but not in the CSC population. In vivo, the ceramide-based nanocarrier showed a prolonged effect, with enhanced antitumor effect and a good safety profile [50]. The idea of using a bioactive compound as building material for drug delivery systems was also explored by Wang et al. who developed a SN-38 nanoprodrug platform loaded with Sali for combinatorial treatment of hepatocellular carcinoma. For constructing the nanoformulations, SN-38, the active metabolite of irinotecan, was modified with linoleic acid, thus allowing it to self-assemble upon injection in an aqueous media. The prodrug nanoparticles were also PEGylated with DSPE-PEG2000, and used as carrier for Sali. This approach allowed the formation of well-defined spherical structures with sizes of approximately 60–70 nm, high encapsulation (over 95%) of both drugs, and slow-release rate. Compared to free Sali and SN-38 nanoprodrug carrier, the co-loaded nanosystem had stronger anti-proliferative, pro-apoptotic and antimigratory effects in hepatocellular carcinoma by efficient elimination of the CSC population. In cell-derived tumor xenograft (CDX) and patient-derived tumor xenograft (PDX) models in mice, even though free Sali failed to suppress tumor growth, the delivery by SN-38 prodrug nanocarrier favored strong antitumor effects by promoting synergism of the two drugs [52].

Inspired by the product Genexol-PM, which is a formulation of paclitaxel in PEG-PLA polymeric micelles, Daman et al. used the same copolymer to construct micellar carriers for the delivery of Sali to gemcitabine-resistant pancreatic cancer. They employed two different methods of preparation, namely the nanoprecipitation and film hydration techniques to fabricate the micelles. While the preparation method had no influence on the entrapment efficiency and drug loading of Sali (which were over 85% and 4%, respectively), a significant effect was observed regarding the size of the micelles. Interestingly, the entrapment of Sali increased the size of the micelles from 30–40 nm to over 100–150 nm, and the negative surface charge, but overall, the nanoprecipitation method produced particles with smaller size, which were able to retain the embedded drug and subsequently release 90% of it in a biphasic pattern, within 48 h. Sali-loaded polymeric micelles were found to induce noticeable antitumor effects in gemcitabine-resistant AsPC-1 pancreatic cancer cells and tumor-bearing mice, by inducing apoptosis, and inhibiting invasion and migration of tumor cells. Surprisingly, the observed cytotoxicity was not significantly different from that of free Sali. However, the higher survival probability of mice treated with micellar Sali compared to the free drug suggests the potential of this polymeric nanocarrier for future applications [91]. Moreover, Zhang et at reported the synthesis of PEG-b-PCL micelles for the separate delivery of Sali and paclitaxel in breast cancer. Besides, the paclitaxel-loaded micelles were further conjugated with Octreotide peptide for the selective binding of somatostatin receptors expressed on MCF-7 cancer cells. The 25 nm-size, spherical Sali-micelles were more effective in suppressing breast CSCs in vivo compared to free Sali. In addition, the combination of Octreotide-modified polymeric micelles containing paclitaxel and Sali-loaded micelles exhibited a strong antitumor effect towards breast cancer cells and CSCs, which was observed in vitro and in tumor-bearing mice [94].

Since multidrug resistance (MDR) is accountable for chemotherapy failure in many types of cancer patients, inhibition of the ABC transporters could facilitate the accumulation of anticancer drugs in tumor tissue. Since Sali is a substrate for P-glycoprotein (P-gp) efflux pump, Sousa et al. proposed the incorporation of this drug in Pluronic F127 micelles for enhanced anticancer effects towards lung cancer. Micellar Sali indeed down-regulated the expression of P-gp leading to an increased intracellular accumulation of the drug, but only after 24 h of exposure. This effect appeared to be time- and dose-dependent since a longer incubation time stimulated the expression of the MDR gene. In order to develop the polymeric micelles, the research group employed the Quality by Design (QbD) approach, and based on two Design of Experiments (DoE) were able to establish an optimal micellar formulation with predefined characteristics. The Pluronic F127 micelles possessed all necessary attributes for the successful delivery of Sali to tumor cells: small size around 26 nm, uniform dispersion, acceptable stability (Zeta potential of −10.7 mV), and excellent entrapment efficiency of 97.9%. The Sali-loaded micelles decreased the migration of A549 lung cancer cells by harnessing the EMT mechanism via down-regulation of mesenchymal VIM protein. Furthermore, micellar Sali displayed antibacterial activity against methicillin-resistant *S. aureus* (MRSA), but not *S. aureus* and *E. coli* which could promote this delivery system for dual anticancer and antimicrobial therapy [95].

Nanomicelles, whether lipidic or polymeric, have showed a lot of promise in recent oncological research. Their effectiveness as drug delivery systems of Sali mainly stems from their small size, narrow particle size distribution, high drug loading, and flexibility in design. As stated earlier in this paper, micelles are made up of amphiphilic block polymers with the ability to self-assemble in contact with an aqueous environment. Various materials have been used to manufacture micelles, especially DSPE-PEG. The PEG layer helps the micelles bypass recognition in the bloodstream. Given these advantages, researchers have extensively exploited micelles as nanocarriers for Sali, more so than any other nanosystem mentioned beforehand. Owing to their small size in the range of 20–30 nm, micelles efficiently mediated the internalization of Sali in cancer cells. The release of the payload at the tumor site is essential for effective eradication of cancer cells. Interestingly, in most instances, the amount of Sali released from micelles was higher at an acidic pH 5.0–5.5 than at physiological pH 7.4. Since the tumor microenvironment is acidic, this allowed the preferential release of the payload at the tumor site. Similar to other types of nanosystems, the surface of micelles can be functionalized with various ligands such as peptides and aptamers, which favored the internalization into target cancer cells compared to naked micelles. One interesting strategy was to use an active drug, methotrexate, as homing ligand to target specific folate receptors. Furthermore, drugs could be conjugated to the polymer or a hydrophobic moiety through a chemical reaction to obtain active polymer chains. Such was the case of methotrexate and SN38 which produced self-assembled constructs which provided an opportunity for combined delivery of Sali for a superior therapeutic outcome in vitro and in vivo. Notably, micellar formulations of Sali were well tolerated in animal models, suggesting that incorporation into micelles could reduce the toxicity of the drug.

### 4.5. Polypeptide- and Protein-Based Nanosystems

Different types of polypeptide- and protein-based nanosystems with Sali have been reported in the literature including drug-conjugates, nanoparticles, and hydrogels (Table 7).

Zhao et al. constructed and characterized an immune-tolerant elastin-like polypeptide (iTEP) delivery system which improved the pharmacokinetic profile and tumor accumulation of Sali in breast cancer. Sali served both as the hydrophobic segment of the iTEP-drug conjugate (via a stable MPBH linker) and the payload of the nanocarrier. The Sali-loaded iTEP-Sali conjugates assumed micelle-like nanoparticle structure with a size of 195 nm and moderate polydispersity (0.288), but low entrapment efficiency (25%) and rapid release rate (around 50% in the first hour). Additional encapsulation of positively charged *N*,*N*-dimethylhexylamine (DMHA) and lipophilic α-tocopherol further improved the encapsulation efficiency and release of Sali to 75% and a half-life of 4.1 h, respectively. Although Sali-loaded iTEP-Sali nanoparticles exhibited similar cytotoxicity towards 4T1 breast CSCs to free Sali, they failed to inhibit tumor growth. It had been suggested that a slower release of Sali from the nanocarriers or a combinatorial approach could promise better results [42]. Therefore, to improve the formulation of the carrier, Zhao et al. synthesized iTEP-Sali conjugates by inserting a cleavable covalent bond for controlled release of Sali. In addition, the amphiphilicity of the conjugate was boosted by modifying Sali with a pH-sensitive linker, 4-(aminomethyl)benzaldehyde (ABA) to generate a more hydrophobic Sali-ABA segment. Compared to the iTEP-Sali carrier from the previously-mentioned study, the novel iTEP-Sali-ABA conjugate displayed a much longer pH-dependent release half-life (12.15 h at pH 5) and reduced size (51.2 nm) which contributed to a prolonged circulation, and enhanced tumor accumulation and cytotoxicity. The iTEP-Sali-ABA nanoparticles inhibited the primary tumor growth and metastasis of 4T1 breast cancer and improved metastasis-free survival and overall survival compared to control; however, the tumor inhibitory effect was insufficient for the stabilization of the primary tumor. In contrast, a combination therapy of iTEP-Sali-ABA conjugate and paclitaxel nanoparticles proved to be more effective than the corresponding monotherapies in inhibiting the primary tumors and prolonging the survival of mice bearing 4T1 orthotopic breast tumors [49].

With the aim to improve the biopharmaceutical properties of Sali and to achieve a targeted action at the tumor site, Awad et al. developed a protein conjugate in which Sali was attached through a sensitive photo linker to trans-activator of transcription (TAT) protein. According to the research group, this association is very stable in physiological conditions, and only harsh conditions like a pH of 12 and a temperature of 75 °C would allow the cleavage of the conjugate and release of Sali. Considering the photo sensitivity of the linker, UV irradiation at a wavelength of 365 nm for 100 s allowed the total release of Sali from the conjugate which could be further translated into an immediate exposure of the cancerous cells to the therapeutic drug. Since this outcome depended on the cellular uptake of the formulation, the conjugate was further associated with an azido sugar moiety, which increased the cytotoxic response on MCF-7 and JIMT-1 human breast cancer cell lines and decreased the IC_50_ value to half [41].

In another study, a novel pharmaceutical formulation was designed by Wu et al. using silk fibroin (SF) extracted from cocoons. According to the authors, SF represents a new excipient in the synthesis of nanoparticles and gels with good biocompatibility. However, the extraction of SF from cocoons is time consuming, and the extraction efficiency was not mentioned. The drugs, paclitaxel and Sali, were first incorporated into SF nanoparticles, and subsequently into the SF gel. The complementary incorporation of the nanoparticles into the gel aimed at obtaining a consistent formulation with respect to drug content, since SF nanoparticles have the tendency to form deposits. Concerning the therapeutic efficacy of the formulation, the in vivo administration of Sali-SF nanoparticles increased the maximum tolerated dose of Sali by doubling it, while the administration of paclitaxel and Sali-SF nanoparticle-gel presented the highest tumor growth inhibitory effect in a murine H22 hepatic model. Moreover, the research group studied the ability of the cancerous cells collected from treated mice to form new tumors in healthy mice. Results showed that the volume of the new formed tumors in paclitaxel and Sali-SF nanoparticle-gel treated group was significantly reduced in comparison with the one in the control group. As the authors underlined, the failure to inhibit the formation of new tumors stems from the fast release of Sali from the formulation, which reached 83% in the first 9 h, while paclitaxel presented a prolonged release over a period of 30 days [96].

Even though there are several papers reporting the effectiveness of Sali-based combination therapy for eradicating both the bulk tumor tissue and the CSC population, most studies focus on the association with conventional chemotherapy agents such as doxorubicin, docetaxel, or paclitaxel, as previously discussed in this paper, but few studies have approached photodynamic therapy with photosensitizing agents as anti-CSC strategy. One study reported the preparation of Sali and chlorin e6 keratin nanoparticles via nanoprecipitation using vitamin E acetate as aggregating agent, for the combined treatment of breast cancer. This association aimed to complement the CSC-specific effects of Sali with the photosensitizing potential of chlorine e6 upon light irradiation. The dual-loaded nanoparticles were highly monodispersed around 127 nm, spherical, with a low negative surface charge (−27 mV), and able to completely release Sali in the first 7 h by non-Fickian diffusion. When loaded in a ratio of 1:1.4, Sali and chlorin e6 exhibited a synergistic effect against MDA-MB-231 and MCF-7 breast cancer cells, allowing to reduce the dose of Sali. However, MCF-7 proved to be less sensitive to the combined treatment. In vitro, the drug combination, especially in nanoparticulate form was able to inhibit the formation of mammospheres and reduce the stemness of breast cancer cells by eliminating CSCs. These effects were correlated with the ability of the nanoparticles to interfere with the Wnt/β-catenin pathway, observed in vivo, in zebrafish embryos [97].

Protein-based nanosystems are attractive carriers since they can be easily manufactured from a variety of natural or engineered polypeptides or proteins derived from different sources such as animals, insects, or recombinant protein bacterial expression systems. What is advantageous about using proteins as drug delivery systems is their biocompatibility, biodegradability, and lack of toxicity. Most polypeptides or proteins are enzymatically metabolized after administration. However, a downside of protein-based nanosystems is the possibility to trigger an immune response, especially by those proteins which are not endogenous to the human body. A feature which makes them unique is the flexibility in chemical modifications due to the abundant functional groups (amino, carboxyl, hydroxyl) in their backbone [98]. Thus, protein-based nanosystems have been proven versatile delivery systems for Sali. Interestingly, polypeptide-based nanosystems show a faster release of Sali (on average under 12 h) compared to other types of nanoparticles, which could hamper the successful delivery of Sali to tumor sites due to rapid loss in the bloodstream or other tissues. However, in most studies presented above, Sali retained its cytotoxicity towards cancer cells, especially CSC-rich cultures, after incorporation into polypeptide-based nanosystems, allowing the decrease in dosage and showing superior effectiveness to the free drug.

### 4.6. Metallic Nanoparticles

Metallic nanoparticles can be obtained from various metals like iron, gold, or silver. Alongside their ability to transport drug molecules at the target site, metallic nanoparticles can also be used for heat-triggered drug release, which can lead to an immediate exposure of the cancerous cells to chemotherapeutic agents, and in this way increase the nanoparticles’ cytotoxic profile [65]. These main benefits of metallic nanoparticles were also exploited to deliver Sali to tumors, and the primary results are summarized in Table 8.

Using gold nanoparticles, Zhao et al. managed to attach Sali on the nanoparticles’ surface via a PEGylated compound, namely thiol-PEG-amine. The physiochemical characterization of the nanoparticles evidenced a mean diameter of approximately 20 nm and an encapsulation efficiency of 63.2%. After Sali’s conjugation, the Zeta potential value increased from −24.8 mV to −4.2 mV, stressing out a decrease in the suspension’s stability. The in vitro experiments demonstrated the pronounced inhibitory effect of Sali-conjugated gold nanoparticles on MCF-7 breast cancer cells as well as on CSCs. Moreover, by adding different inhibitory components (apoptosis, necrosis or ferroptosis inhibitors) in co-treatment with free Sali or Sali-conjugated gold nanoparticles, the research group confirmed that cell death occurred via ferroptosis, one of Sali’s mechanism of action [40]. Following the same objectives, Xu et al. developed gold nanorods conjugated with Sali for potential applications in breast cancer therapy. Compared to other types of nanoparticles which usually are spherical in shape, nanorods presented an elongated shape with a length of 56 nm and a height of 16 nm. In this case, nanorods were developed with the aim to release drug in a temperature dependent manner, a quality attribute supported by the in vitro release study which evidenced a two-fold increase in the total percentage of released drug at 48 °C in contrast to 37 °C. The influence of temperature on drug release was also noted in the cytotoxicity profile, cell viability decreasing to less than 20% when the co-treatment between gold nanorods conjugated with Sali and laser irradiation were applied, versus approximately 90% for Sali-conjugated nanorods alone [99].

In another study, Norouzi et al. attached Sali at the surface of iron oxide nanoparticles (IONPs) for the treatment of glioblastoma. For this purpose, IONPs were conjugated with PEG to improve their biopharmaceutical properties and with polyethylene imine (PEI) to be able to attach Sali to the nanoparticles’ surface. The IONPs’ were monodispersed with a hydrodynamic diameter of 84 nm but an encapsulation efficiency of only 3.45%. As it was previously observed, the attachment of Sali at the surface of metallic nanoparticles influenced the Zeta potential, in this case decreasing it from +27.14 mV to +0.8 mV. The cytotoxic effect of the nanoparticles was found to be similar to that of free Sali on U251 glioblastoma cell line, but it could be increased in a blood–brain barrier–glioblastoma in vitro model when a magnetic field and a 2% mannitol solution were used. Moreover, the previously-mentioned external factors also enhanced the permeability of the nanoparticles from 1% to 3.2% [100].

The unique magnetic, electronic and optical properties of metallic nanoparticles make them highly useful for various biomedical applications, including oncology. Metallic nanoparticles exhibit increased cytotoxicity due to their small size (below 100 nm) and increased surface area which is amenable for functionalization. Generally, metallic nanoparticles have been reported as physically and chemically stable, biocompatible and environmentally friendly. However, their size, shape, surface charge, and functionalization decide their toxicity. Overall, metallic nanoparticles have proven their efficacy as carriers for Sali to tumor sites [65].

### 4.7. Nanotubes

Carbon nanotubes are the most common type of carbon-based nanoparticle with biomedical applications. They are composed of carbon, bearing the shape of a tube, and can be divided into two classes: single-walled carbon nanotubes (SWCNTs) and multi-walled carbon nanotubes (MWCNTs). Like metallic nanoparticles, carbon nanotubes possess unique structural, physical, chemical, electrical, and thermal features. Their quality attributes are highly important for their cytotoxic profile [65]. In this view, Yao et al. attached Sali at the surface of SWCNTs, followed by a double coating with chitosan and hyaluronic acid with the aim to improve SWCNT hydrophilicity and to prevent their fast elimination. The nanoparticle’s characterization evidenced a polydisperse suspension (PDI 0.34), the mean diameter of functionalized SWCNTs being 234 nm, while the drug loading was 20.96%. The in vitro release studies highlighted a pH-triggered release controlled by chitosan; the total percentage of drug released was 60% at pH 5.5 vs. 20% at pH 7.4. The role of hyaluronic acid was to target CD44+ gastric CSCs, thus promoting the cell internalization of SWCNTs. This behavior was very well underlined in the proliferation studies, SWCNTs loaded with Sali and coated with hyaluronic acid being more noxious on CSCs, while on regular cancerous cells the nanotubes presented a significantly reduced toxicity compared to free Sali [101].

The drug combination between Sali and paclitaxel seems to be a preferred option for the in vitro/in vivo treatment of breast cancer, as it was already mentioned in this paper. Considering this, Faraj et al. attached these two active substances on SWCNTs using 4-hydrazinobenzoic acid (HBA) for attaining a pH-dependent drug release. To improve the therapeutic and pharmacokinetic properties, the nanotubes were functionalized with PEG and with CD44 monoclonal antibodies. The functionalization of the nanoparticles together with the combination of the two drugs proved to be an effective strategy for the treatment of MDA-MB-231 breast cancer tumor-bearing mice. Treatment with the co-loaded nanotubes caused a reduction of the tumor volume of forty times compared to the control group. However, the in vitro experiments evidenced that single drug-loaded nanotubes inhibited cell proliferation depending on the drug’s potency on the cell type, paclitaxel being more potent on the regular cell line, while Sali on CSCs. This suggests that for a successful therapeutic outcome, carbon nanotubes need to contain both drugs [102].

Carbon nanotubes are gaining more ground as drug delivery systems. Their large surface area and chemical stability, enables them to conjugate various drugs [65], including Sali (Table 8). However, issues related to their potential toxicity remain a highly debated topic and reason for serious concern, which may limit their use as drug carriers irrespective of their proven efficacy.

### 4.8. Other Types of Drug Delivery Nanosystems

Nanoparticles can be synthesized from different types of materials, and as a result will display different quality attributes that make them suitable for different applications [103]. Various other types of nanosystems for the delivery of Sali are reported in Table 9. Regardless of their production method or type of material used for their synthesis, the main goal of nanoparticles as drug carriers is to deliver drugs at the target site with the purpose of increasing patient compliance, reducing adverse reactions and improving therapeutic responses [104].

In light of this, Liang et al. developed a prodrug of Sali using D-α-tocopheryl succinate. This new prodrug in association with PEGylated D-α-tocopheryl succinate and hyaluronic acid conjugate were able to form nanoparticles with sizes around 200 nm. The main quality of this new formulation was the redox sensitivity, which enabled the nanoparticles to release the entrapped drug in the presence of elevated concentrations of glutathione which is normally found in cancerous cells and in the tumor microenvironment. The total percentage of drug released was 94% vs. 15% (in the absence of glutathione) within 48 h. The ability of the nanoparticles to penetrate MCF-7 spheroids was observed microscopically; hyaluronic acid allowed a deeper access of the nanoparticles and a disruption of the spheroids’ integrity due to a targeted action [2].

In a recent study, Norouzi et al. incorporated Sali into PLGA nanofibers using electrospinning as the preparation technique. Nanofibers presented a diameter of 170 nm and a stability of four days, after which a constant degradation process was observed within four weeks due to PLGA’s low stability. The total percentage of drug released was 80% in the first four days, followed by a sustained release up to 10 days. Concerning the antiproliferative properties on U251 glioblastoma cell line, it was noted that Sali nanofibers inhibited cell proliferation to a higher degree compared to free Sali when the entrapped drug concentration reached 1 µg/mL. This result was explained by the fact that Sali presented a prolonged release from the nanofibers [105]. With the aim to achieve the same therapeutic outcome, Norouzi et al. developed in another study a thermosensitive hydrogel loaded with Sali using Pluronic F-127 and PLGA-PEG-PLGA triblock copolymer as the main excipients. In this case a differentiated cytotoxic effect between the two formulations was observed on U251 glioblastoma cell line, the Pluronic hydrogel exhibiting a greater inhibitory effect compared to the PLGA-PEG-PLGA hydrogel [106].

Taking into account the biocompatibility and other benefits of lipids when used for targeted drug delivery systems, Zhou et al. developed nanostructured lipid carriers loaded with Sali to target stem cells from non-small cell lung cancer. To ensure the success of the formulation, TISWPPR peptide was attached at the surface of the nanoparticles to actively target NCI-H1299 stem cells by binding to the CD133 marker. The encapsulation efficiency of Sali reached 95%, while the in vitro studies revealed a four-times higher cytotoxicity of the formulation compared to free Sali [107]. In another study, solid lipid nanoparticles loaded with Sali and coated with clathrin were developed to prevent the premature drug release during blood circulation time and to achieve a burst release into cancerous cells. To demonstrate that the objective was accomplished, release studies were performed in different media and dynamic conditions, i.e., still plasma, cytoplasm ± HSC70 protein, ultra-sound agitated plasma, or various filtering flow rates. These studies revealed that the total percentage of Sali released from clathrin-coated nanoparticles was minimized compared to uncoated nanoparticles, except for the medium that contained HSC70 protein which deteriorated the clathrin shell and allowed a burst release of the drug. Moreover, the incorporation of fluorescent compounds into the nanoparticles revealed the high uptake of clathrin-coated nanoparticles by HepG2 human liver cancer cells and explained the increased cytotoxic effect of the referred nanoparticles compared to the uncoated nanoparticles or free Sali [108]. Considering the benefits of drug combination in cancer treatment, Tsakiris et al. used excipients approved by the FDA to develop lipid nanocapsules co-loaded with Sali and SN-38. The physiochemical characterization of the nanoparticles evidenced an encapsulation efficiency of nearly 100% for Sali and 72% for SN-38, while the particle size was 50 nm with a PDI lower than 0.1. This study showed that the incorporation of Sali into lipid nanocapsules can increase the in vivo tolerability by four times, compared to its free form. Furthermore, the co-loaded lipid nanocapsules reduced the tumor volume in a murine HCT116 colorectal cancer model and increased the median survival time [109].

By appealing to different chemical techniques, Liénard et al. synthesized bis-triazolium-based cyclopolylactides functionalized with folic acid or rhodamine, which in aqueous solution precipitate and form nanoparticles with the ability to incorporate Sali. The encapsulation efficiency of Sali reached 79% and 84% in both types of functionalized nanoparticles, but a great difference was observed between the nanoparticles’ size, a value close to 385 nm being observed for the folic acid-functionalized nanoparticles and a value close to 125 nm for the rhodamine-functionalized nanoparticles. The viability studies were performed on MG-63 human osteosarcoma spheroids, which revealed a pronounced cytotoxic effect of both loaded and unloaded functionalized nanoparticles [110].

## 5. Clinical Perspectives

Until now, Sali has been used in clinical practice with therapeutic purposes only in the veterinary area [8], but as the number of in vitro/in vivo studies performed on different cancer cell lines with promising results increases, the interest of using this drug in human anticancer therapeutic schemes accentuates. In this context, Naujokat and Steinhart presented two case reports where Sali was used as an experimental drug to treat triple-negative breast cancer and squamous cell carcinoma of the vulva in patients with recurrences and no other therapeutic options. Clinical investigations proved that Sali helped in preventing tumor progression/recurrence and decreased the concentration of tumor markers. Despite this, Sali therapy after intravenous administration in concentrations of 200 or 250 µg/kg induced acute side effects in both patients, namely tachycardia and a mild tremor for a short period of time [8].

Except for these data, there are other studies that evidenced the systemic toxicity of Sali [46,111], suggesting the need to incorporate this compound into nanoparticles to increase patient compliance and achieve a targeted action.

Considering the studies that have been cited in this review, we may conclude that the most promising nanoparticles with future perspectives in cancer therapy (Figure 4) are polymeric nanoparticles and micelles for breast cancer, liver cancer, or osteosarcoma therapy. Since these studies represent the foundation for new product development, Hillstream BioPharma designed a polymeric nanosystem with Sali (HSB-1216) for the treatment of small cell lung cancer and triple negative breast cancer. The novel QUATRAMER technology entails the incorporation of Sali in polymeric nanoparticles composed of PEG-polypropylene glycol (PPG)-PEG-modified PLA tetra-block copolymer [112,113,114,115]. Preclinical studies highlighted that in the case of lung cancer, once/week administration of the formulation in a xenograft model at a dose of 5 mg/kg inhibited the tumor growth with no evident side effects, while the in vitro studies performed on MDA-MB-231 triple negative breast cancer cell line evidenced a near total inhibition of mammosphere formation compared to paclitaxel by selectively binding to the CD44 marker [112,113,116]. These positive results constituted the basis of planning a phase I clinical trial in patients with metastatic small cell lung cancer that followed or are following a cancer therapy with platinum-based drugs [117].

## 6. Conclusions and Future Perspectives

Cancer is a serious health issue, having a large contribution to the total mortality registered worldwide. In recent research, CSCs have been proven to play a pivotal role in the pathogenesis of cancer, having the ability to initiate and perpetuate different tumors. Since CSCs are highly resistant to conventional chemotherapeutic agents and radiation therapy, it is necessary to find new strategies for treating various types of cancer. Sali, an ionophore antibiotic, has emerged as a promising new anticancer agent with strong inhibitory effects on different types of cancer cells, including CSCs and multidrug-resistant cancer cells. The selective cytotoxic activity of Sali against CSCs may bring research one step closer to finding an appropriate cure for cancer. However, promise comes with limitations. The inappropriate physicochemical and pharmacokinetic properties of Sali renders it relatively unsuitable for clinical use in humans. The formulation in nanosized carriers has been proven to be an effective means of overcoming current limitations of Sali. Several in vitro and in vivo studies performed on Sali-loaded nanoplatforms have demonstrated the much-improved therapeutic efficiency compared to free Sali. Although liposomes are the most common and well-investigated nanosystems for drug delivery, and already numerous products have been approved for cancer therapy, only a small number of studies regarding liposomal Sali are available. It seems that scientific interest has shifted towards other types of nanocarriers, mainly polymeric nanoparticles and micelles. The higher stability of polymeric nanoparticles compared to liposomes have made them attractive drug delivery systems for Sali. Moreover, nanomicelles are gaining popularity mainly because of their small size which favors the accumulation of anticancer drugs at tumor sites.

However, in spite of the notable efforts and breakthrough of nanomedicine in fighting cancer, currently there is limited success in the clinical translation of such drug delivery systems. No clinical trials involving Sali-loaded nanoparticles have been performed in humans yet. One reason for this might by the unsatisfactory targeting ability of the nanosystems, since only a fraction of the administered dose effectively reaches the tumor site. The fundamental principle of passive targeting relies on the EPR effect, which in turn is controlled by the specific features of tumors. The heterogenous nature of tumors is largely responsible for the significant variability in therapeutic response in patients. However, active targeting, which uses various ligands and moieties to selectively target specific receptors or molecules, has been validated as more effective in tackling tumors. Therefore, a deeper understanding of the pathophysiology and molecular biology of cancer could steer scientists towards a rational design of formulations. Other limitations for the implementation of Sali nanoparticles into clinical practice may stem from the changes in the physical properties of the nanoparticles, i.e., size, PDI, and Zeta potential, that occur during the preparation process. These quality attributes are essential for ensuring the EPR effect or a prolonged blood circulation time, thus a successful therapeutic response. Moreover, the disparity between the types of nanoparticles, preparation techniques and loading methods led to variability in Sali’s encapsulation efficiency. The identification of a proper preparation method is critical since the toxicity of the nanosystem depends on the amount of encapsulated drug, and may as well reduce the production costs, administered dose/dose intervals.

Furthermore, since it has been postulated that the conversion between CSCs and differentiated cancer cells is essential for tumors to survive and disseminate, the combination of Sali (an anti-CSC drug) and a standard chemotherapy agent (which kills fast dividing cells) could ensure the elimination of different subpopulations of cancer cells. Combination therapy is, therefore, advantageous if the two anticancer agents act thorough distinct mechanisms and do not induce potentiated toxicity. Moreover, Sali has been proven effective in overcoming MDR by sensitizing cancer cells to standard anticancer agents as a result of P-glycoprotein inhibition. Based on these, the incorporation of such a therapeutic combination in the same nanocarrier or separately has been proven even more effective in eradicating both regular cancer cells and CSCs. Paclitaxel is the most frequently used conventional anticancer agents in association with Sali in nanosystems. The association of two distinct nanosystems, each containing a therapeutic drug, is a simpler approach, but is mainly limited by the difficulty of maintaining a synergistic effect because of the specific pharmacokinetic profile of each nanocarrier, and by the different quality attributes (size, PDI, Zeta potential) of the nanoparticles. In addition, the dosing protocol might raise some concern among the medical staff, since special attention must be paid to the administered dose of each type of nanosystem, while the patient compliance might diminish. The co-delivery approach, even though is more advantageous in terms of dosing regimen, has proven to be more challenging. Apart from being more complex in terms of design, maintaining a synergistic drug ratio in the same nanosystem is not easily achieved since each drug exhibits a different release rate depending on their characteristics. This is particularly the case when Sali, a hydrophobic drug, is associated with a hydrophilic agent such a doxorubicin.

Clearly, a rational design and further optimization of the formulations of these nanoplatforms and future research is needed to achieve a favorable therapeutic response in cancer.

## Figures and Tables

**Table 1:** The advantages and disadvantages of different types of nanoparticles used for the drug delivery of Sali.

Type of Nanoparticle	Advantages	Disadvantages	Ref.
Liposomes	- Biocompatible/biodegradable/non-immunogenic/non-toxic; - can incorporate hydrophilic and lipophilic drugs; - can incorporate various compounds, including enzymes and genetic material; - the formulation can be adjusted for a specific delivery route; - multifunctional/smart liposomes can be developed.	- The entrapped drug concentration is dependent on the internal volume of the liposomes; - low encapsulation efficiency.	[55,56,57]
Polymeric nanoparticles	- Numerous biomedical applications; - can entrap hydrophilic and lipophilic drugs; - biodegradable/biocompatible; - high encapsulation efficiency/drug loading.	- Some polymers are rapidly degraded or possess a poor solubility in numerous solvents; - the scale-up process is challenging; - nanoparticles obtained from synthetic polymers might cause environmental concerns.	[55,58,59]
Polymer–lipid hybrid nanoparticles	- The use of combined excipients can lead to biocompatible nanoparticles with low cytotoxic profile, improved stability and increased in vivo activity; - are able to deliver more than one active substance.	-	[60]
Micelles	- biocompatible; - possess prolonged blood circulation time/release profile; - the surface can be functionalized with ligands and peptides; - stimuli-sensitive nanoparticles can be obtained; - uniform in size.	- Can incorporate only lipophilic drugs; - the incorporation of the active substances depends on the interaction with the excipients.	[55,56,61]
Polypeptide/protein-based nanoparticles	- Can incorporate hydrophilic and lipophilic drugs; - the surface can be modified with ligands; - can cross the blood–brain barrier; - the induced immune response is reduced in comparison with other types of nanoparticles; - easy manufacturing process and scale-up.	- Different proteins tend to have different affinity for hydrophilic/lipophilic molecules.	[62,63,64]
Carbon Nanotubes	- Can deliver drugs/genetic material/proteins; - multiple biomedical applications.	- Poor solubility in water; - can induce inflammatory reactions in different organs.	[55,56,65]
Metallic nanoparticles	- Can deliver more than one drug; - the surface properties can be easily modified during the preparation process; - non-immunogenic; - multiple biomedical applications.	- Lack of information regarding their toxicity and biopharmaceutic properties; - non-biodegradable.	[40,55,65]

**Table 2:** Liposomal formulations with salinomycin.

Composition	Payload	Preparation Method	Size (nm)	PDI	Zeta Potential (mV)	EE (%)	Drug Loading (%)	In Vitro Release	Biological Effect	Ref.
DPPC, CHOL (2:1), DSPE-PEG2000 (5 mol%)	Sali complexes (Na, K, Ni, Co, Mn)	Lipid film hydration method, freeze-drying	133.8–159.9 ^1^	0.062–0.124 ^1^	+0.4–+4.04 ^1^	28–76 ^1^	n.r.	n.r.	Divalent complexes were more cytotoxic; liposomal complexes were more effective than the free form in KG-1, Reh and U266 cells	[4]
HSPC, CHOL, DSPE-PEG2000 (85:10:5)	Sali + Dox	Lipid film method	115	0.215	−41.1	68.34/52.06 ^2^	1.31/0.88 ^2^	80% in PBS pH 7.4/70% in PBS pH 5, at 12 h ^2^	Synergistic effect at 1:1 molar ratio; the liposomal combination exhibited a higher tumor inhibitory rate, and CSC-eradicating effect compared to free combination in HepG2 tumor-bearing mice	[66]
DOPC, DOPG, MPB-PE (4:1:5), PEGylated	Sali + Dox	Dehydration-rehydration method, crosslinking with DTT	265	0.027	n.r.	> 80 (for both drugs)	n.r.	80%/70% in media containing 10% FBS, after 15 days ^2^	Higher inhibitory effect of CSCs for the liposomal combination than the single liposomal drugs in 4T1, 4T1D and MDA-MB-231 cells. 2-fold more effective in vivo that the single liposomal drugs or their combination	[67]
HSPC, CHOL, DSPE-PEG2000 (85:10:5)	Sali+Cq	Ethanol injection method	120.9	0.174	−13.7	68.62/60.97 ^3^	2.60/8.33 ^3^	80%/40% in PBS pH 7.4, at 12 h ^3^	Synergistic effect at 1:5 molar ratio; Cq enhanced the cytotoxicity of Sali in HepG2 cells	[68]

PDI, polydispersity index; EE, entrapment efficiency; DPPC, 1,2-dipalmitoyl-sn-glycero-3-phosphocholine; CHOL, cholesterol; DSPE-PEG2000, 1,2-distearoyl-sn-glycero-3-phosphoethanolamine (methoxy(polyethylene glycol)-2000); Sali, salinomycin; n.r., not reported; HSPC, hydrogenated soybean phospholipid; Dox, doxorubicin; CSC, cancer stem cell; DOPC, 1,2-dioleoyl-sn-glycero-3-phosphocholine; DOPG, 1,2-dioleoyl-sn-glycero-3-phospho-(10-rac-glycerol); MPB-PE, 1,2-dioleoyl-sn-glycero-3-phosphoethanolamine-*N*-[4-(p-maleimidophenyl)butyramide]; PEG, polyethylene glycol; DTT, dithiothreitol; FBS, fetal bovine serum; Cq, chloroquine. ^1^ Data reported for various liposomal salinomycin complexes, depending on complex:DPPC molar ratio. ^2^ Data reported for salinomycin and doxorubicin, respectively. ^3^ Data reported for salinomycin and chloroquine, respectively.

**Table 3:** Polymeric nanoparticles with salinomycin.

Composition	Preparation Method	Size (nm)	PDI	Zeta Potential (mV)	EE (%)	Drug Loading (%)	In Vitro Release	Biological Effect	Ref.
PLGA	Emulsion diffusion evaporation method	187.4	0.11	+51.0	97.4	n.r.	43% in PBS pH 7.4 + 0.3% sodium azide, at 24 h; 100% after 45 days	Decreased the proliferation and enhanced the apoptosis of MG-63 cells	[10]
PCL, modified with PEGylated gelatinase-responsive peptide (PVGLIG)	Single emulsion method	n.r.	n.r.	n.r.	89.70	n.r.	n.r.	Inhibitory effects against HeLa CSCs, in vivo; reduced toxicity compared to free Sali	[51]
PLGA, conjugated with EGFR and CD133 aptamers	Emulsion/solvent evaporation method	118.3–152.8 ^1^	0.13–0.18 ^1^	−23.3-(−34.7) ^1^	51.5–58.1 ^1^	7.0–9.3 ^1^	80% in PBS pH 7.4 and 90% in rat plasma, after 500 h	Greater antitumor effect against HCC of dual conjugated NPs in vitro and in vivo, compared to single-conjugated or unconjugated NPs	[53]
PLGA, conjugated with CD133 aptamers	Emulsion/solvent evaporation method	133.4/159.8 ^2^	0.13/0.15 ^2^	−23.6/−30.1 ^2^	55.9/53.1 ^2^	7.2/6.8 ^2^	50% in PBS pH 7.4 and human plasma, at 24 h; 85% after 12 days	Aptamer-conjugated Sali-NPs were 5 and 2-fold more effective against Saos-2 CD133^+^ cells than Sali-NPs and Sali; selective cytotoxicity against CD133^+^ CSCs in vitro and in vivo	[54]
PCL, modified with PEGylated gelatinase-cleavable peptide (PVGLIG)	Nanoprecipitation and single emulsion methods	151.1/235.8 ^3^	0.099/0.160 ^3^	n.r.	81.51/89.70 ^3^	7.40/8.12 ^3^	79%/70% in PBS pH 7.4, at 24 h ^3^	Higher survival rate of mice compared to free Sali	[70]
PLA, functionalized with folate	Nanoprecipitation method	110/875.0 ^4^	n.r.	n.r.	98/99 ^4,5^	8.8/8.9 ^4,5^	n.r.	No difference in cytotoxicity against MG-63 cell compared to free Sali; superior anti-CSC effect for folate-decorated NPs in CSC-enriched culture	[71]
PLGA, coated with Polysorbate 80	Solvent emulsion-evaporation method	195.3–293.6 ^6^	0.259–0.423 ^6^	n.r.	57.2–62.9 ^6^	n.r.	63.5–95.4% in PBS pH 7.4 + 0.1% sodium azide, after 480 h ^6^	Greater targeting ability and decrease in cell viability of T98G cells for Polysorbate 80-coated NPs compared to naked NPs	[72]
PLGA, decorated with Herceptin	Solvent emulsion-evaporation method	194.9–257.5 ^7^	0.024–0.297 ^7^	n.r.	61.3–91.7 ^7^	1.62–19.0 ^7^	32.9–79.6% in PBS pH 7.4 + 0.1% sodium azide, after 360 h ^7^	Herceptin immobilization favored cellular uptake of Sali-NPs in MCF7 cells	[73]
PLGA-PEG, conjugated with CD133 antibody	Emulsion/solvent evaporation method	139.9/149.2 ^8^	0.16/0.18 ^8^	−19.6/−22.8 ^8^	68.3/63.2 ^8^	9.9/8.5 ^8^	45% in PBS pH 7.4 and PBS + 10% FBS, at 24 h; 80% after 12 days	Enhanced cytotoxicity and anti-CSC effect against OVCAR-3 and PA-1 cells and in vivo antitumor efficacy of antibody-conjugated Sali-NPs, compared to naked Sali-NPs and free Sali	[74]
PLGA-TPGS	Nanoprecipitation method	62.86	0.21	−28.7	56.35	4.79	n.r.	Higher Sali solubility and oral bioavailability by incorporation into NPs; superior efficacy against NC stem cells in tumor-bearing mice, compared to free Sali	[77]

PDI, polydispersity index; EE, entrapment efficiency; PLGA, poly(lactic-co-glycolic acid); n.r., not reported; PCL, polycaprolactone; PEG, polyethylene glycol; CSC, cancer stem cell; Sali, salinomycin; EGFR, epidermal growth factor receptor; HCC, hepatocellular carcinoma; NP, nanoparticle; PLA, poly(lactic acid); FBS, fetal bovine serum; TPGS, d-α-tocopherol polyethylene glycol succinate; NC, nasopharyngeal carcinoma. ^1^ Data reported for unconjugated, single aptamer-conjugated and dual aptamer-conjugated salinomycin-loaded nanoparticles, respectively. ^2^ Data reported for native and CD133 aptamers-functionalized salinomycin-loaded nanoparticles, respectively. ^3^ Data reported for the nanoprecipitation method and single emulsion method, respectively. ^4^ Data reported for native and folate-functionalized salinomycin-loaded nanoparticles, respectively. ^5^ Data measured by gel permeation chromatography. ^6^ Data reported for different nanoparticle formulations, naked or coated with Polysorbate 80, and prepared with different salinomycin concentrations (5 µM and 10 µM). ^7^ Data reported for different nanoparticle formulations, naked or decorated with Herceptin, and prepared with different salinomycin concentrations (1 µM and 15 µM). ^8^ Data reported for naked and CD133 antibody-conjugated salinomycin-loaded nanoparticles, respectively.

**Table 4:** Combined delivery of salinomycin and different anticancer drugs in polymeric nanoparticles.

Composition	Combination Therapy	Preparation Method	Size (nm)	PDI	Zeta Potential (mV)	EE (%)	Drug Loading (%)	In Vitro Release	Biological Effect	Ref.
PLGA-PEG	Sali + Docetaxel ^1^	Emulsion/solvent evaporation method	129.4 ^2^	0.11 ^2^	−17.3 ^2^	79.2 ^2^	7.4 ^2^	50% in PBS pH 7.4 and human plasma, at 12 h; 80%, after 108 h	Superior tumor growth suppression of GC compared to combined free drugs and single drug-NPs	[75]
PLGA-TPGS	Sali + Docetaxel ^3^	Nanoprecipitation method	73.83	0.193	−25.7	53.28/82.3 ^4^	4.08/4.12 ^4^	68.19%/65.43% in PBS pH 5.0, and 64.28%/60.52% in PBS pH 7.4, after 10 days ^4^	Synergistic effect at 1:1 molar ratio; greater cytotoxicity in MCF-7 cells and mammospheres, and superior antitumor efficacy in vivo, compared to other treatments	[76]
PLGA-PEG	Sali + Gefitinib ^5^	Emulsion/solvent evaporation method	146.8/132.5 ^6^	0.13/0.15 ^6^	−16.3/−18.8 ^6^	83.8/76.3 ^6^	8.7/7.5 ^6^	80% after 108 h; faster release in PBS + 10% FBS than in PBS pH 7.4 ^6^	Effective eradication of CD133+ lung CSCs and inhibition of tumorsphere formation; the combination of drug-loaded NPs inhibited tumor growth in A431-xenograft-bearing mice more efficiently than the combined free drugs or single drug-loaded NPs; good safety profile in vivo	[78]
PLGA, coated with HA	Sali + Paclitaxel ^7^	Emulsion solvent diffusion method	153.41/116.71 ^8^	0.258/0.257 ^8^	+49.1/+68.2 ^8^	71.2/59.7 ^8^	10/5 ^8^	100%/60% in PBS pH 7.4 + 0.5% Tween 80, after 60 days ^8^	Sali’s cytotoxicity increased by 2.3 and 5.7-fold by incorporation into NPs and HA-NPs, respectively; HA coating of NPs improved cellular uptake by 1.5-fold; combination of NPs showed the highest potency against CD44^+^ breast cancer cells	[79]
PLGA-PEG, conjugated with HA	Sali + Curcumin ^9^	Double emulsion method	153.4/120.1 ^10^	n.r.	n.r.	70/82 ^11^	n.r.	88%/90% in PBS pH 7.4 and 96%/94% in PBS pH 5.0, at 24 h	Higher efficacy in inducing apoptosis and inhibiting cell migration of MCF-7 cells	[80]

PDI, polydispersity index; EE, entrapment efficiency; PLGA-PEG, poly(ethylene glycol)-b-poly(lactic-co-glycolic acid) copolymer; Sali, salinomycin; GC, gastric cancer; NP, nanoparticle; TPGS, d-α-tocopherol polyethylene glycol succinate; FBS, fetal bovine serum; PLGA, poly(lactic-co-glycolic acid); HA, hyaluronic acid; n.r., not reported. ^1^ Salinomycin and docetaxel were incorporated into nanoparticles separately. ^2^ Data reported for salinomycin-loaded nanoparticles. ^3^ Salinomycin and docetaxel were co-loaded in the nanoparticles. ^4^ Data reported for salinomycin and docetaxel, respectively. ^5^ Salinomycin and gefitinib were incorporated into nanoparticles separately. ^6^ Data reported for salinomycin-loaded nanoparticles and gefitinib-loaded nanoparticles, respectively. ^7^ Salinomycin and paclitaxel were incorporated into nanoparticles separately. ^8^ Data reported for hyaluronic acid-coated salinomycin-loaded nanoparticles and paclitaxel-loaded nanoparticles, respectively. ^9^ Salinomycin and curcumin were co-loaded in the nanoparticles. ^10^ Data reported for naked co-loaded nanoparticles and hyaluronic acid-conjugated co-loaded nanoparticles, respectively. ^11^ Data reported for salinomycin and curcumin, respectively.

**Table 5:** Polymer–lipid hybrid nanoparticle formulations with salinomycin.

Composition	Preparation Method	Size (nm)	PDI	Zeta Potential (mV)	EE (%)	Drug Loading (%)	In Vitro Release	Biological Effect	Ref.
PLGA, soybean lecithin, DSPE-PEG2000, conjugated with anti-HER2 Fab’	Nanoprecipitation method	123.2/135.6 ^1^	0.12/0.15 ^1^	−25.6/−28.3 ^1^	59.2/55.4 ^1^	8.8/8.0 ^1^	50% in PBS and PBS + 10% FBS, at 24 h; 80% in PBS and 90% in PBS + 10% FBS, after 96 h	Encapsulation in NPs promoted cellular delivery of Sali; greater cytotoxicity against HER2-positive breast CSCs and cancer cells, in vitro and in vivo, compared to unconjugated NPs and free Sali	[81]
PLGA, phosphatidylcholine, DSPE-PEG, CHOL (57:3:40), conjugated with EGFR and CD133 Fab’	Emulsion-solvent evaporation method	107.8	0.18	−14.4	78.1	9.3	60% in PBS and PBS + 10% FBS, at 24 h; 80% after 96 h	Encapsulation in NPs facilitated the cellular delivery of Sali; dual-targeted NPs were more effective against lung cancer than untargeted NPs, single-targeted NPs and free Sali	[82]
PLGA, soybean lecithin, DSPE-PEG, conjugated with CD20 aptamers	Nanoprecipitation method	92.1/96.3 ^2^	0.12/0.11 ^2^	-20.3/-20.9 ^2^	69.4/61.8 ^2^	7.9/7.8 ^2^	60% in PBS and 70% in PBS + 10% FBS, at 24 h; 80% after 96 h	Lower IC_50_ and increased tumor growth inhibition of melanoma CSCs compared to unconjugated NPs and free Sali	[83]
PLGA, phosphatidylcholine, DSPE-PEG, CHOL (57:3:40), conjugated with CD44 Fab’	Emulsion-solvent evaporation method	125.6/139.9 ^3^	0.13/0.17 ^3^	−13.4/−17.3 ^3^	76.3/74.2 ^3^	8.1/8.9 ^3^	45% in PBS and PBS + 10% FBS, at 24 h; 80% after 120 h	Specific delivery of Sali to prostate CSCs and greater inhibition of CSCs than unconjugated NPs and free Sali	[84]
PLGA, soybean lecithin, DSPE-PEG, conjugated with EGFR aptamer	Nanoprecipitation method	89.6/95.6 ^4^	0.12/0.11 ^4^	-21.6/-26.4 ^4^	66.7/63.1 ^4^	7.8/8.9 ^4^	50% in PBS pH 7.4 and PBS + 10% FBS, at 24 h; 80% after 120 h	Significantly more effective towards osteosarcoma CSCs than unconjugated NPs and free Sali	[85]
PLGA, soybean lecithin, DSPE-PEG, conjugated with CD133 and EGFR aptamers	Solvent emulsion diffusion method	110.2	0.15	−17.7	66.5	9.4	60% in PBS and PBS + 10% FBS, at 24 h; 80% after 72 h	3- to 7-fold higher cytotoxicity in osteosarcoma cells and CSCs and significant decrease in tumor growth in osteosarcoma-bearing mice, compared to untargeted NPs, Sali-NPs and free Sali	[86]
PLGA, soybean lecithin, DSPE-PEG2000, conjugated with GE11 peptide	Nanoprecipitation method	132.6 ^5^	n.r.	-51.2 ^5^	n.r.	n.r.	n.r.	3-fold greater cellular uptake and suppression of cell migration for targeted NPs compared to nontargeted NPs in MCF-7 cells; GE11-conjugated Sali-NPs had higher cytotoxic effect against MCF-7 cells in vitro than nontargeted NPs, but similar to free Sali; strongest tumor inhibitory effect in vivo for GE11- conjugated Sali-loaded NPs, compared to controls	[88]

PDI, polydispersity index; EE, entrapment efficiency; PLGA, poly(lactic-co-glycolic acid); DSPE-PEG2000, 1,2-distearoyl-sn-glycero-3-phosphoethanolamine-N- (methoxy(polyethylene glycol)-2000); FBS, fetal bovine serum; NP, nanoparticle; Sali, salinomycin; CSC, cancer stem cell; CHOL, cholesterol; EGFR, epidermal growth factor receptor; n.r., not reported. ^1^ Data reported for unconjugated and anti-HER2 antibody-conjugated salinomycin-loaded nanoparticles, respectively. ^2^ Data reported for unconjugated and CD20 aptamers-conjugated salinomycin-loaded nanoparticles, respectively. ^3^ Data reported for unconjugated and CD44 antibody-conjugated salinomycin-loaded nanoparticles, respectively. ^4^ Data reported for unconjugated and EGFR aptamer-conjugated salinomycin-loaded nanoparticles, respectively. ^5^ Data reported for GE11-conjugated salinomycin-loaded nanoparticles.

**Table 6:** Micellar formulations with salinomycin.

Composition	Combination Therapy	Preparation Method	Size (nm)	PDI	Zeta Potential (mV)	EE (%)	Drug Loading (%)	In Vitro Release	Biological Effect	Ref.
DSPE-PEG2000, conjugated with iRGD	-	Lipid film method	14.0/13.7 ^1^	0.24/0.31 ^1^	−17.7/−17.1 ^1^	96.6/93.4 ^1^	9.1/8.9 ^1^	80% in PBS pH 5.5 + 0.1% SDS and 60% in PBS 7.4 + 0.1% SDS, at 48 h	Increased cytotoxicity against HepG2 cells and tumorspheres, compared to untargeted NPs and free Sali, respectively; superior penetration in tumor and efficacy in liver cancer-bearing mice	[48]
PEG-ceramide	-	Lipid film method	14.6	0.25	−4.4	76.7	6.3	75% in PBS pH 5.0 + 0.1% SDS and 50% in PBS pH 7.4 + 0.1% SDS, at 12 h	Synergistic effects of Sali and PEG-ceramide at 1:4 molar ratio; 4.5 and 2-fold increase in cytotoxicity in HepG2 cells and tumorspheres, compared to free Sali, at 48 h; increased apoptosis in HepG2 cells, but not in tumorspheres, compared to Sali; good safety profile and higher tumor growth inhibitory effects in vivo, compared to Sali	[50]
DSPE-PEG2000	LA-SN38 prodrug ^2^	Injection method	61.7	n.r.	n.r.	97.24/99.98 ^3^	32.71/33.64 ^3^	80%/70% in PBS pH 7.4 + 0.1% Tween 80, at 96 h ^3^	Synergistic effect; Sali reduced the IC_50_ of SN38 in HCC; stronger apoptotic effect in HCC cells, compared to free Sali and SN38 prodrug NPs; increased anti-CSC effect and decreased migration and invasion of HCC cells, compared to Sali; significant decrease in tumor volume in vivo, compared to Sali	[52]
DSPE-PEG2000	MTX ^4^	Lipid film method	21.8	0.15	−21.2	83.1/85.7 ^5^	9.0/5.4 ^5^	85% in PBS pH 5.5 and 70% in PBS pH 7.4, at 48 h/20% in PBS pH 5.5 and PBS pH 7.4, at 48 h ^5^; protease-dependent release profile	Enhanced inhibitory effects against HNSCC CSCs and in tumor-bearing mice, compared to single-loaded NPs and free drugs; significant reduction in toxicity of free drugs in mice	[89]
PLA-PEG2000	-	Nanoprecipitation method and film hydration method	127.1/154.5 ^6^	0.22 ^7^	n.r.	85.6–90.2 ^7^	4.8–8.7 ^8^	90% in PBS pH 7.4 + 0.5% SDS, at 48 h	Significant toxicity in AsPC-1 cells and tumor inhibition, but similar to free Sali; higher survival probability in tumor-bearing mice	[91]
DSPE-PEG2000, conjugated with EGFR aptamer	-	Lipid film method	22.4/24.3 ^9^	0.16/0.18 ^9^	−19.5/−19.7 ^9^	82.1/80.3 ^9^	10.4/9.7 ^9^	70% in PBS and 80% in PBS + 10% FBS, after 72 h	Increased cell penetration and cytotoxicity of micellar Sali in lung cancer cells and CSCs, compared to free Sali; higher antitumor efficacy of EGFR-targeted micelles in vivo, compared to untargeted NPs and free Sali	[93]
PCL-PEG	PTX ^10^	Film hydration method	27.21	0.13	n.r.	99.78	n.r.	97.9% in PBS + 0.5% SDS, at 24 h	Micellar Sali effectively suppressed breast CSCs in vitro and in vivo; Sali sensitized PTX against MCF-7 cells; the combination of micellar Sali and Oct-modified PTX-NPs was more effective in vivo, compared to the single drug NPs or combined free drug treatments	[94]
Pluronic F127	-	Film hydration method	26 ^11^	0.22 ^11^	−10.7 ^11^	97.9 ^11^	n.r.	n.r.	No statistical difference in cytotoxicity against A549 cells between micellar Sali and free Sali; significant reduction in cell migration of A549 cells, compared to free Sali; time- and dose-dependent effect on P-gp expression; higher antibacterial activity towards MRSA than free Sali	[95]

PDI, polydispersity index; EE, entrapment efficiency; DSPE-PEG2000, 1,2-distearoyl-sn-glycero-3-phosphoethanolamine-*N*-(methoxy(polyethylene glycol)-2000); SDS, sodium dodecyl sulfate; NP, nanoparticle; Sali, salinomycin; PEG, polyethylene glycol; LA-SN38, linoleic acid conjugated (7-ethyl-10-hydroxycamptothecin); n.r., not reported; HCC, hepatocellular carcinoma; CSC, cancer stem cell; MTX, methotrexate; HNSCC, head and neck squamous cell carcinoma; PLA, poly(lactic acid); EGFR, epidermal growth factor receptor; FBS, fetal bovine serum; PCL, polycaprolactone; PTX, paclitaxel; Oct, octreotide; SDS, sodium salicylate; P-gp, P-glycoprotein; MRSA, methicillin-resistant *Staphylococcus aureus*. ^1^ Data reported for unconjugated and iRGD-conjugated salinomycin-loaded micelles, respectively. ^2^ Salinomycin and SN38 prodrug were co-loaded in the micelles; SN38 was formulated as linoleic acid-SN38 prodrug nanoparticles. ^3^ Data reported for salinomycin and SN38 prodrug, respectively. ^4^ Methotrexate and salinomycin were co-loaded in the micelles; methotrexate was conjugated to DSPE-PEG2000. ^5^ Data reported for salinomycin and methotrexate, respectively. ^6^ Data reported for the nanoprecipitation method, for drug to polymer ratios of 5% and 10%, respectively. ^7^ Data reported for micelles obtained by the nanoprecipitation method, with a drug to polymer ratio of 10%. ^8^ Data reported for the nanoprecipitation and film hydration methods, and for different drug to polymer ratios (5% and 10%). ^9^ Data reported for unconjugated and EGFR aptamer-conjugated salinomycin-loaded micelles, respectively. ^10^ Paclitaxel was loaded in octreotide-modified PCL-PEG micelles, separately from salinomycin. ^11^ Data reported for the optimal formulation of salinomycin-loaded micelles.

**Table 7:** Polypeptide- and protein-based nanosystems with salinomycin.

Composition	Combination Therapy	Preparation Method	Size (nm)	PDI	Zeta Potential (mV)	EE (%)	Drug Loading (%)	In Vitro Release	Biological Effect	Ref.
TAT protein	-	Conjugation through a photosensitive linker; attachment of solubilizing sugar moiety by click chemistry	n.r.	n.r.	n.r.	n.r.	n.r.	Complete release upon irradiation at ≥ 365 nm within 80–100 s.	More that 4-fold reduction in IC_50_ values by conjugation with TAT protein in MCF-7 and JIMT-1 breast cancer cells, compared to free Sali	[41]
Elastin-like polypeptide (iTEP), DMHA, α-tocopherol	-	Conjugation through a chemical reaction	179.9	n.r.	+0.046	75.4	n.r.	100% in PBS pH 7.4, at 24 h	Similar cytotoxicity with free Sali in 4T1 mammospheres; slower clearance and 2.4-fold greater tumor accumulation in vivo, but lower accumulation in heart and lung, than free Sali; 1.1-fold reduction in CSC frequency in tumor-bearing mice	[42]
Elastin-like polypeptide (iTEP)	PTX ^1^	Conjugation ^2^ through a chemical reaction	85.09	n.r.	n.r.	84.6 ^3^	n.r.	Half-life of 12.15 h in 0.1 M sodium acetate- acetic acid buffer pH 5.0/4.67 h in PBS pH 7.4 ^4^	30-fold increase in AUC, 35-fold increase in elimination half-life and 3.4-fold increase in tumor accumulation by incorporation into NPs than free Sali; greater inhibition of primary 4T1 breast tumor and metastasis by Sali-ABA NPs compared to free drug; the combination therapy with PTX slowed down tumor growth and improved overall survival of mice more efficiently	[49]
Silk fibroin	PTX ^5^	Nanoprecipitation, ultrasound-induced cross-linking ^6^	241.0 ^7^	0.147 ^7^	−14.24 ^7^	34.7 ^7^	12.1 ^7^	94.5% in PBS, at 24 h, 98.3%, after 5 days/17.5% in PBS + 0.5% Tween 80, after 30 days ^7,8^	Reduction in Sali toxicity by incorporation into NP; locoregional dual drug SF gel administration produced smaller tumors in H22 tumor-bearing mice, compared to systemic administration of dual drug SF gel and single drugs; effective anti-CSC effect in vivo; dual drug SF gel showed superior tumor growth inhibition effect and longer survival of mice than other treatments	[96]
Keratin, vitamin E acetate	Ce6	Nanoprecipitation	127	0.13	−27	n.r.	n.r.	100% in PBS pH 6.8 + Tween 80, after 7 h	Synergistic effect in MCF-7 and MDA-MB-231 breast cancer cells; reduction in Sali dosage; incorporation into keratin NPs reduced mammosphere formation efficiency, compared to free drugs	[97]

PDI, polydispersity index; EE, entrapment efficiency; TAT, trans-activator of transcription protein; n.r., not reported; Sali, salinomycin; DMHA, *N*,*N*-dimethylhexylamine; CSC, cancer stem cell; PTX, paclitaxel; AUC, area under the curve; NP, nanoparticle; SF, silk fibroin; Ce6, chlorin e6. ^1^ Salinomycin and paclitaxel were co-loaded into the nanoparticles. ^2^ Salinomycin was modified by conjugation with a pH-sensitive linker (4-(aminomethyl)benzaldehyde, ABA), yielding salinomycin-ABA. ^3^ Data reported for paclitaxel. ^4^ Data reported for salinomycin-ABA and paclitaxel, respectively. ^5^ Paclitaxel was loaded into silk fibroin nanoparticles separately from salinomycin. ^6^ Data reported for the preparation of silk fibroin nanoparticles and nanoparticle-loaded silk fibroin gel, respectively. ^7^ Data reported for salinomycin-loaded silk fibroin nanoparticles prepared with a silk fibroin concentration of 15 mg/mL and salinomycin amount of 6 mg. ^8^ Data reported for salinomycin and paclitaxel, respectively (from the hydrogel).

**Table 8:** Metallic nanoparticles and carbon nanotubes with salinomycin.

Composition	Combination Therapy	Preparation Method	Size (nm)	PDI	Zeta Potential (mV)	EE (%)	Drug Loading (%)	In Vitro Release	Biological Effect	Ref.
Metallic nanoparticles with salinomycin										
Gold, SH-PEG-NH_2_	-	Sodium citrate reduction method	20.9	n.r.	−4.2	n.r.	63.2	n.r.	More pronounced inhibitory effect compared to free Sali; Sali induces an increase of ROS production; NPs cause cell death through ferroptosis	[40]
Gold	-	Seed-mediated silver-assisted approach; electrostatic adsorption	56 × 16	n.r.	53.6	n.r.	22.6	Approximatively 20% at 48 °C and less than 10% at 37 °C after 24 h;Maximum 7% in PBS pH 7.4 after irradiation	Irradiation promotes Sali release which leads to a synergistic effect and a more pronounced inhibitory effect. After 15 min of irradiation, cell viability decreased to less than 20% while the viability of ALDH^+^ cells decreased to almost 0%	[99]
Iron (III) acetylacetonate, PEI, PEG	-	Chemical reactions	84.1	0.132	0.8	3.45	n.r.	Sustained release for 72 h; pH 4.5 favors the release of Sali (66%) compared to pH 7.4 (44%) in the first hours	Similar toxicity with free Sali on U251 cell line; cell uptake of NPs was concentration-dependent; the application of a magnetic field favored NP uptake; the permeability of NPs was increased when a magnetic field and a 2% mannitol solution were applied in a blood–brain barrier-GB in vitro model	[100]
Carbon nanotubes with salinomycin										
SWCNT, conjugated with HA and chitosan	-	Non-covalent functionalization	154.55/200.13/237.09 ^1^	0.26/0.38/0.34 ^1^	−28.77/+2.56/−11.23 ^1^	n.r.	32.74/26.29/20.96 ^1^	< 20% in PBS pH 7.4 in 48 h ^1^; 60% in PBS pH 5.5 in 12 h ^2^	HA favored the cell uptake of NPs through CD44 receptor; SWCNT functionalized with chitosan and HA exhibited the greatest inhibitory effect on CSCs	[101]
SWCNT-PEG; 4-hydrazinobenzoic acid, conjugated with CD44 antibodies	PTX ^3^	n.r.	n.r.	n.r.	n.r.	n.r.	1.8 mg of Sali/1 mg of SWCNT; 1.7 mg of PTX/1 mg of SWCNT	50% in PBS pH 5.5 in 12 h for Sali or in 18 h for PTX	Synergistic effect between Sali-SWCNT and PTX-SWCNT on MDA-MB-231 cells; the co-treatment with Sali-SWCNT and PTX-SWCNT reduced the tumor volume by 40 times	[102]

PDI, polydispersity index; EE, entrapment efficiency; SH-PEG-NH_2_, tiol-polyethylene glycol-amine; n.r., not reported; Sali, salinomycin; ROS, reactive oxygen species; NP, nanoparticle; PEI, polyethylenimine; PEG, polyethylene glycol; GB, glioblastoma; SWCNT, single-wall carbon nanotube; HA, hyaluronic acid; CSC, cancer stem cell; PTX, paclitaxel. ^1^ Data reported for salinomycin-loaded SWCNT, chitosan-functionalized salinomycin-loaded SWCNT, chitosan and hyaluronic acid-functionalized salinomycin-loaded SWCNT, respectively. ^2^ Data reported for chitosan-functionalized salinomycin-loaded SWCNT and chitosan and hyaluronic acid-functionalized salinomycin-loaded SWCNT, respectively. ^3^ Paclitaxel was loaded into nanotubes separately from salinomycin.

**Table 9:** Other types of nanoparticles loaded with salinomycin.

Composition	Combination Therapy	Preparation Method	Size (nm)	PDI	Zeta Potential (mV)	EE (%)	Drug Loading (%)	In Vitro Release	Biological Effect	Ref.
TPGS, HA, D-α-tocopheryl succinate	PTX	Covalent linkage; Emulsion-solvent evaporation method	203/193/280/238 ^1^	0.18/0.15/0.24/0.15 ^1^	−6.77/−29.4/−6.81/−28.87 ^1^	92.61/87.67 ^2^	3.08/2.92 ^2^	> 90% in PBS pH 6.8 + Tween 80 and glutathione ^3^	3-fold decrease of IC_50_ value for the HA-coated NPs after 72 h treatment exposure	[2]
PLGA	-	Electrospinning	170 ^4^	n.r.	n.r.	n.r.	n.r.	80% in PBS pH 6 and 7.4, after 4 days	A more pronounced inhibitory effect compared to free Sali; induction of ROS production	[105]
Pluronic F-127, PLGA-PEG-PLGA triblock co-polymer	-	n.r.	n.r.	n.r.	n.r.	n.r.	n.r.	Pluronic hydrogel was degraded in one week while PLGA-PEG-PLGA hydrogel in one month in PBS pH 7.4 at 37 °C; 100%/36% in PBS pH 7.4, after 7 days ^5^	Both types of hydrogels presented a higher cytotoxic effect compared with free Sali; both types of hydrogels loaded with Sali induced ROS production	[106]
ATO-5, MCT, Solutol HS15, Kolliphor EL, polyoxyethylene 40 stearate, DSPE-PEG2000, conjugated with TISWPPR peptide	PTX ^6^	Melt emulsification and solidification method	128.73	n.r.	-28.3	95.62	1.02	95% in PBS pH 7.4 + ethanol and SDS, after 24 h	The inhibitory effect of the NPs was 2-times higher compared to free Sali, and 4-times higher when TISWPPR peptide was attached to the surface of the NPs	[107]
Soybean lecithin, CHOL, modified with clathrin	-	Film hydration method	199/310 ^7^	n.r.	-33.53/-36.63 ^7^	97.96/93.5 ^7^	12.81/10.14 ^7^	The release rates in still plasma and still cytoplasm were similar, 11.8% and 6.5%, respectively; in still cytoplasm with HSC70 was approximately 11.8%; in ultrasounded plasma was 15.1/8.8% ^7^. All the experiments were performed for 72 h	Clathrin-modified NPs exhibited a higher inhibitory effect compared to free Sali or Sali-SLN when used in concentrations greater than 2.5 µM	[108]
Labrafac, Trancutol, Lipoid S-100, Solutol HS15	SN38 ^8^	Phase inversion temperature method	54.2	0.08	−1.3	100	24.1	n.r.	The encapsulation into NPs reduced the hemolytic activity and IC_50_ value, increased the tolerated dose in vivo and median survival time	[109]
c-PLA, azido-folate, azido-rhodamine	-	Chemical synthesis; click coupling; nanoprecipitation method	385/125 ^9^	n.r.	n.r.	79/84 9	7.1/7.6 9	n.r.	Folate-decorated NPs presented an increased cytotoxic effect, and the incorporation of Sali into NPs did not improve this outcome; Sali-loaded NPs and decorated with rhodamine increased the cytotoxic effect of the unloaded NPs or free Sali	[110]

PDI, polydispersity index; EE, entrapment efficiency; TPGS, D-α-tocopheryl polyethylene glycol 1000 succinate; HA, hyaluronic acid; PTX, paclitaxel; NP, nanoparticle; PLGA, poly(lactic-co-glycolic acid); n.r., not reported; Sali, salinomycin; ROS, reactive oxygen species; PLGA-PEG-PLGA, poly(lactic-co-glycolic acid)-polyethylene glycol-poly(lactic-co-glycolic acid) triblock co-polymer; MCT, medium chain triglyceride; DSPE-PEG2000, 1,2-distearoyl-sn-glycero-3-phosphoethanolamine (methoxy(polyethylene glycol)-2000); SDS, sodium dodecyl sulfate; SLN, solid lipid nanocarriers; CHOL, cholesterol; c-PLA, cyclicpolylactide. ^1^ Data reported for salinomycin-loaded nanoparticles, hyaluronic acid-conjugated salinomycin-loaded nanoparticles, salinomycin and paclitaxel-loaded nanoparticles, hyaluronic acid-conjugated salinomycin and paclitaxel-loaded nanoparticles, respectively. ^2^ Data reported for paclitaxel loaded in the naked nanoparticles and hyaluronic acid-conjugated nanoparticles, respectively. ^3^ Data reported for salinomycin and paclitaxel, respectively. ^4^ The size refers to the diameter of the nanofibers. ^5^ Data reported for Pluronic F-127 hydrogel and PLGA-PEG-PLGA hydrogel, respectively. ^6^ Paclitaxel was loaded into the nanoparticles separately from salinomycin. ^7^ Data reported for salinomycin-loaded solid lipid nanocarriers and clathrin-modified salinomycin-loaded solid lipid nanocarriers, respectively. ^8^ SN38 was loaded into the nanoparticles separately from salinomycin. ^9^ Data reported for folate-functionalized salinomycin-loaded nanoparticles and rhodamine-functionalized salinomycin-loaded nanoparticles, respectively.
